# Single-cell sequencing reveals dysregulated cell type perturbations and critical mediator communication remodelling in colorectal cancer

**DOI:** 10.3389/fimmu.2025.1557564

**Published:** 2025-06-05

**Authors:** Chengyuan Xu, Siqi Zhang, Zhouyu Zhang, Luoqiu Zhang, Bin Sun, Zicheng Yu, Hailong Liu

**Affiliations:** ^1^ Department of General, Yangpu Hospital, School of Medicine, Tongji University, Shanghai, China; ^2^ Center for Clinical Research and Translational Medicine, Yangpu Hospital, School of Medicine, Tongji University, Shanghai, China; ^3^ Department of Pharmacy, Yangpu Hospital, School of Medicine, Tongji University, Shanghai, China

**Keywords:** single-cell sequencing, colorectal cancer, tumour microenvironment, cellular heterogeneity, immune regulation, TUBB

## Abstract

**Background:**

The heterogeneity of colorectal cancer (CRC) and its complex immune microenvironment pose significant challenges for treatment. Understanding the cellular composition and dynamic changes is essential for uncovering mechanisms of tumour progression.

**Methods:**

To investigate the cellular heterogeneity and immune microenvironment of CRC, identifying critical subpopulations, functional pathways, and prognostic biomarkers, single-cell transcriptomic data from 41 CRC samples across four datasets were integrated. Bioinformatic analyses identified cellular subpopulations, cell communication networks, and prognostic biomarkers. The expression patterns, clinical significance and biological function of *TUBB* were validated *in vitro*.

**Results:**

A distinct epithelial subpopulation with proliferative and invasive features was identified, promoting tumour progression by resisting apoptosis and remodelling the extracellular matrix. ActMono, a terminal state of myeloid cells, was enriched in tumours and linked to disease progression. Cell communication analysis highlighted galectin signalling in immune regulation. A prognostic model (CRS) based on secretory immune cell-related genes identified *TUBB* as a key molecule influencing the cell cycle and extracellular matrix remodelling, with its expression patterns, clinical significance and biological effects validated *in vitro*.

**Conclusion:**

This study reveals critical subpopulations, signalling pathways, and biomarkers in CRC, providing insights into tumour progression and potential therapeutic strategies.

## 1 Introduction

Colorectal cancer (CRC) ranks as the third most common malignancy globally and represents a leading cause of cancer-related mortality. Despite surgical resection combined with chemotherapy being the standard treatment protocol, approximately one-third of patients experience disease recurrence (1, 2). While immune checkpoint inhibitors have shown significant efficacy in microsatellite instability-high (MSI-H) tumours and combined EGFR/BRAF inhibitor therapy has proven effective in BRAF V600E-mutant CRC, these treatments are only applicable to specific patient subgroups (3–5). Large-scale gene expression studies have established molecular classification systems for CRC, most notably the Consensus Molecular Subtypes (CMS), which categorizes CRC into four subtypes: CMS1**–**4 (6). However, these classifications, which are primarily based on bulk sequencing data, cannot precisely resolve the complex cellular heterogeneity within the tumour microenvironment.

The development of CRC involves the accumulation of mutations in multiple oncogenes and tumour suppressor genes (such as *APC, KRAS*, and *PIK3CA*) and microsatellite instability caused by DNA mismatch repair gene dysfunction (3, 7). Although high tumour mutational burden (TMB) and MSI status can predict the response to immune checkpoint inhibitor therapy, only a minority of patients respond to PD-1 inhibitor treatment (8, 9). The complex molecular heterogeneity and microenvironmental characteristics of CRC not only influence disease progression but also present significant challenges for precision medicine, highlighting the importance of understanding the CRC microenvironment in detail. To date, there has not been a comprehensive and systematic characterization of how tumour and TME cells shape the tumoural, stromal, and immune landscapes to form specific CRC subtypes.

Recent single-cell studies have revealed cellular heterogeneity in the CRC microenvironment and identified multiple functionally important specific cell subgroups (10–13). While these studies have provided new perspectives for understanding tumour progression mechanisms and immune evasion, their geographical limitations and sample sizes make fully characterizing the shared mechanisms within the CRC microenvironment difficult. Cross-study comparisons are also challenging due to varying cell annotation methods across different studies. In this study, we integrated four datasets from public databases, encompassing 41 samples, to systematically describe the differential cell population distributions and intercellular interaction networks between tumour and normal tissues. We not only revealed the heterogeneous characteristics and transcriptional reprogramming of epithelial cells in tumour tissues but also identified several key cell subgroups potentially involved in the formation of an immunosuppressive microenvironment, providing new insights into CRC progression mechanisms and the immune microenvironment.

## 2 Materials and methods

### 2.1 Data collection

Data for TCGA-COAD and TCGA-READ were downloaded from the UCSC Xena platform (https://xenabrowser.net/datapages/). Single-cell RNA sequencing (scRNA-seq) datasets were obtained from the GEO database (accession numbers: GSE161277, GSE200997, GSE221575, and GSE231559). The combined dataset included samples from 27 primary colorectal cancer (CRC) patients and 14 normal control samples. After quality control (QC), a total of 88,212 high-quality single cells were retained for further analysis.

### 2.2 Data processing

Single-cell RNA-seq data were preprocessed using the Seurat v4.3.0 R package. Quality control (QC) was performed to remove low-quality cells and potential dying cells. Specifically, we retained cells that expressed at least 400 genes, and excluded cells with >20% mitochondrial gene expression. These thresholds were selected based on the distribution of QC metrics and previous studies, aiming to balance data completeness with the removal of low-quality cells (14–16).

To detect and remove potential doublets, we applied DoubletFinder v2.0.3. The expected number of doublets was calculated based on an assumed doublet rate of ~7.5–8%, following 10X Genomics guidelines, and using the formula: nExp_poi = round(0.08 × N × N/10000), where N is the number of cells in the sample. For doublet prediction, we used 20 principal components (PCs = 1:20) and the following parameters: pN = 0.25, pK = 0.09, nExp = nExp_poi, reuse.pANN = FALSE, sct = FALSE. These settings were based on the recommended defaults in the official DoubletFinder tutorial. Predicted doublets were removed from the dataset prior to downstream analysis.

Data normalization, identification of highly variable genes, principal component analysis (PCA), and unsupervised clustering were performed using Seurat’s standard pipeline. Harmony v1.2.3 was used for batch correction and data integration, with sample identity specified as the batch variable. The RunHarmony() function was executed with default parameters, and the top 30 PCs were retained for downstream analysis. UMAP was used for dimensionality reduction and visualization.

For differential expression analysis, we used the FindAllMarkers() function in Seurat with the Wilcoxon rank-sum test, applying the following thresholds: min.pct = 0.25, logfc.threshold = 0.25, only.pos = FALSE. Significant differentially expressed genes (DEGs) were defined as those with p-value < 0.05.

### 2.3 Cell type identification

To identify cell types, we first performed differential expression analysis across clusters using the FindAllMarkers() function in Seurat. Marker genes were defined as those with an adjusted p-value < 0.05, expression in more than 25% of cells within the cluster (min.pct = 0.25), and an absolute log2 fold change > 0.25. For each cluster, the top-ranked differentially expressed genes were considered cluster-specific highly expressed genes.

We then compared these cluster-specific markers against curated reference databases, including CellMarker and PanglaoDB, to determine the most likely cell type identity for each cluster. Annotation was conducted manually based on the expression patterns of canonical lineage markers and known cell-type-specific genes.

To support and cross-validate our manual annotations, we additionally employed the SingleR package, which uses reference transcriptomic datasets to infer cell identities. The results from SingleR were used as a secondary reference and were reconciled with our primary marker-based annotation strategy.

Furthermore, we calculated Spearman correlation coefficients between the average expression profiles of all clusters to evaluate transcriptional similarity. Clusters with highly correlated expression patterns and overlapping marker gene expression were considered for subtype merging to avoid artificial over-segmentation. Final cell type labels were determined by integrating information from marker gene analysis, database matching, SingleR prediction, and inter-cluster correlation.

### 2.4 Cell communication analysis

Cell-cell communication networks within the tumour microenvironment were inferred using the CellChat v1.1.3 R package based on receptor-ligand interactions (17). Communication probability and the number of interactions were calculated to construct communication networks. The interactions between any two cell populations were visualized, and scatter plots were generated to display the major signalling senders (signal sources) and receivers (targets) in a two-dimensional space, helping to identify the main contributors of outgoing or incoming signals, particularly among immune cell types. A pattern recognition approach was used to identify how multiple immune cell types and signalling pathways coordinate.

### 2.5 Pseudotime trajectory analysis

Pseudotime trajectories were constructed using the Monocle 2 algorithm, an R package designed for single-cell trajectory analysis by Qiu et al. (18). This algorithm reduces high-dimensional gene expression profiles into a low-dimensional space and arranges the cells into trajectories with branching points. Dynamic expression heatmaps were constructed using the plot_pseudotime_heatmap function.

### 2.6 Machine learning-based feature signature identification

A consensus feature signature was derived using a machine learning-based integrative approach that combined ten different algorithms: Random Survival Forest (RSF), Elastic Net (Enet), Lasso, Ridge, Stepwise Cox, CoxBoost, Partial Least Squares for Cox (plsRcox), Supervised Principal Components (SuperPC), Generalized Boosted Regression Model (GBM), and Survival Support Vector Machines (survival-SVM). To ensure robustness, a consensus model was constructed by integrating predictions from these methods.

A total of 101 algorithmic combinations were executed within a leave-one-out cross-validation (LOOCV) framework, optimizing feature selection and model performance. Hyperparameters for each algorithm were tuned using grid search/random search (or specify your method), and model performance was evaluated based on the concordance index (C-index) and other relevant metrics (e.g., AUC, log-rank test).The TCGA-READ and COAD datasets were randomly split into a training set (60%) and a validation set (40%), ensuring a balanced distribution of clinical and molecular features. Stratified sampling was applied to maintain consistency between groups. The final model was assessed on the validation set for predictive accuracy and generalizability.

### 2.7 Immune infiltration evaluation

The CIBERSORT algorithm was applied to quantify immune cell infiltration levels in pancreatic adenocarcinoma (PAAD) patients and to explore differences in immune cell abundance between high-risk and low-risk patient groups (19). Pearson correlation analysis was conducted to assess the relationship between immune cell abundance and risk scores. To further investigate potential differences in immune function, single-sample gene set enrichment analysis (ssGSEA) was employed to obtain enrichment scores, and the Wilcoxon test was used to compare immune function between high-risk and low-risk groups (20).

### 2.8 Quantitative real-time PCR and western blotting

Total RNA extracted from paired colorectal cancer tissues was collected. Then the RNA was reversely transcribed into cDNA with the kit (Takara, Dalian, China) and amplified. The sequences of the primers are detailed as below: *TUBB*: 5’-ATTTCTTTATGCCTGGCTTTG-3’ and 5’-GACCTGCTGGGTGAGTTCC’; *GAPDH*: 5’-ACACCCACTCCTCCACCTTT-3’ and 5’-TTACTCCTTGGAGGCCATGT-3’.

Total cellular protein from clinical samples were lysed with RIPA lysis buffer (Solarbio, China) and added with protease inhibitor at a ratio of 1:100 (Thermo Scientific, United States). The information of primary antibodies was as follows: *TUBB* (1:1,000, Abmart, TA7011M) and *GAPDH* (1:4,000, Abmart, P30008S).

### 2.9 Kaplan–Meier plotter database analysis

We analysed the predictive value of *TUBB* in CRC using Kaplan–Meier (KM) Plotter (https://kmplot.com) (21). Based on the median expression (high and low expression), patients were divided into two groups to analyse the overall survival (OS) and recurrence-free survival (RFS).

### 2.10 Patients and clinical samples

59 paired colorectal cancer tissues were obtained from patients who underwent colorectal cancer surgery at Yangpu Hospital of Tongji University between November 2018 and November 2019. The study got approval from Ethics Committee of the Yangpu Hospital (LL-2023-LW-012). CRC tissues and paracancerous tissues were collected during surgery and immediately frozen in liquid nitrogen to assess the expression levels of specific genes and proteins respectively.

### 2.11 Cell culture and transfection

Human colorectal (CRC) cell lines (HCT116 and SW620) were acquired from Shanghai Institute of Biochemistry and Cell Biology. All cell lines were cultured in DMEM medium (Gibco, Carlsbad, CA, USA) which contains 10% foetal bovine serum (FBS; Gibco) at 37°C with 5% ­CO2.

Lipofectamine 3000 (Invitrogen, Carlsbad, CA, USA) was used to transfect cells with an siRNA specific for *TUBB* and a control construct purchased from GeneChem (Shanghai, China). Cells were utilized for downstream assays at 48h post-transfection. Analyses were conducted in triplicate. *TUBB* overexpression plasmid was customized from GenePharma (Shanghai, China).

### 2.12 Transwell assays and wound healing assay

Cells were suspended in 250 μL of serum-free medium and seeded into the upper chamber of a 24-well Transwell plate (Nest, China). The lower chamber was filled with culture medium containing 10% FBS. For invasion assays, the Transwell chambers were coated with Matrigel (2 mg/mL) and DMEM, whereas for migration assays, they were left uncoated. After 24 hours of incubation, the invaded cells were fixed with 4% paraformaldehyde for 30 minutes and stained with crystal violet for 10 additional minutes, both at room temperature. Cells were counted in five random optical fields of view under a light microscope (Nikon Corporation, Japan).

For the wound healing assay, cells were cultured without FBS in 6-well plates for 24 hours. Linear wounds were created by scratching with a 10 μL pipette tip. Wound closure was monitored and photographed at 0 and 24 hours using a microscope (Nikon Corporation, Japan).

### 2.13 Assays of cell proliferation and apoptosis

To assess the rate of DNA synthesis, CRC cell lines were subjected to treatment with 5-ethynyl-2’-deoxyuridine (EDU) at a concentration of 50 μM, which was subsequently added to the cell culture plates. Following a 30-minute incubation, DNA was stained using Hoechst 33342, allowing for the visualization of positively stained cells under a microscope. HCT116 and SW620 cells, characterized by either *TUBB* overexpression or knockdown, were dissociated into single-cell suspensions using 0.25% trypsin. These cells were then stained with Annexin V-APC and 7-Aminoactinomycin D (7-AAD) to evaluate apoptosis rates, which were quantified through flow cytometry analysis.

### 2.14 Statistical analysis

All statistical analyses and data visualizations were performed using R software (version 4.1.3). Pearson correlation coefficients were used to evaluate the correlation between continuous variables. For quantitative data, a two-tailed unpaired Student’s t-test or one-way analysis of variance (ANOVA) with Tukey’s multiple comparison test was performed to compare values between subgroups. When multiple comparisons were conducted, p-values were adjusted using the Benjamini-Hochberg (BH) method to control the false discovery rate (FDR). A p-value or adjusted p-value < 0.05 was considered statistically significant.

## 3 Results

### 3.1 Integrated analysis reveals cell type composition and functional remodelling in the CRC microenvironment

**Figure 1:**
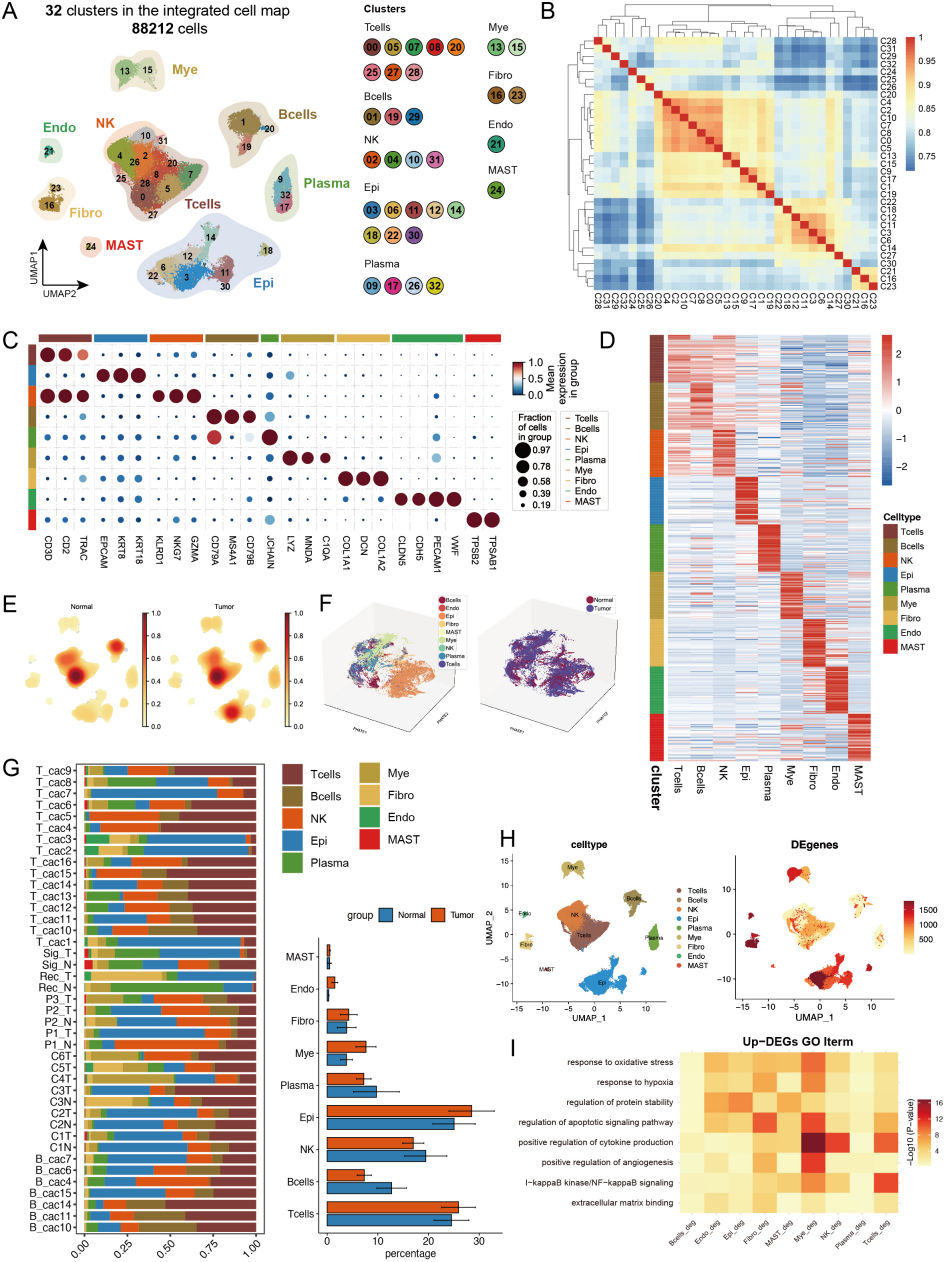
Single-cell transcriptomic analysis reveals cell type composition and functional remodelling in colorectal cancer. **(A)** UMAP dimensionality reduction showing the integrated cell distribution map. A total of 32 cell clusters were identified, classified into 8 major cell types, with different colours representing distinct cell clusters. **(B)** Spearman correlation heatmap based on gene expression across cell clusters, revealing transcriptional similarities between clusters. **(C)** Expression profiles of characteristic marker genes across different cell types. **(D)** Heatmap displaying the top 50 highly expressed genes specific to each cell type, illustrating the transcriptional characteristics of different cell types. **(E)** Density distribution plot of each cell type in tumour and normal groups, showing differences in cell abundance between groups. **(F)** PHATE dimensionality reduction plot displaying cell distribution, with the left panel coloured by cell type and the right panel coloured by sample group, revealing spatial distribution patterns of cell types and sample groups. **(G)** Left panel: Stacked bar plot showing the proportional distribution of different cell types across samples; right panel: Composition ratio of tumour and normal groups within each cell type. * indicates statistical significance at p < 0.05. **(H)** Statistical summary of the number of differentially expressed genes (DEGs) between tumour and normal groups for each cell type. **(I)** Representative GO functional pathways enriched in upregulated DEGs in each cell type.

In this study, we performed an integrated analysis of 41 samples from four public datasets (GSE161277, GSE200997, GSE221575, and GSE231559) (2, 22–24), comprising 27 primary CRC tumour samples and 14 normal control samples. After rigorous quality control and doublet removal, we obtained 88,212 valid cells for analysis (**Supplementary Figures S1A, B**). Through single-cell transcriptome analysis, which combines specific gene expression profiles and classical cell markers, we classified these cells into 32 distinct clusters. On the basis of intercluster Spearman correlation analysis and previously reported cell markers (14, 25), we identified 8 different cell types (**Figures 1A, B**, **Supplementary Figure S1C**), including T cells expressing high levels of *CD3D, CD3E*, and *TRAC*; B cells expressing *CD79A, MS4A1*, and *CD79B*; epithelial cells expressing *EPCAM, KRT8*, and *KRT18*; plasma cells expressing *JCHAIN* and *SDC1*; myeloid cells expressing *LYZ, MNDA*, and *C1QA*; fibroblasts expressing *COL1A1*, *DCN*, and *COL1A2*; endothelial cells expressing *CLDN5, CDH5, PECAM1*, and *VWF*; and mast cells expressing *TPSB2* and *TPSAB1* (**Figure 1C**, **Supplementary Figures S1D–G**). We calculated the top 50 highly expressed genes for each cell type to confirm cluster specificity and used AUCell to score activated pathways in each subgroup, further validating cell identities (**Figure 1D**, **Supplementary Figure S2A**).

For the cell composition analysis, we first calculated the cell type abundance in the tumour and normal groups and found significant enrichment of epithelial cells in the tumour group (**Figure 1E**). Using the PHATE algorithm (26), which captures both local and global nonlinear structures, we identified significant differences between tumour and normal epithelial cells that were not fully revealed by conventional UMAP analysis (**Figure 1F**, **Supplementary Figure S2B**). The cell proportion statistics revealed significant intergroup heterogeneity among the samples. Additionally, the proportions of epithelial cells, and myeloid cells increased in the tumour group, whereas the proportions of B cells and plasma cells decreased, suggesting remodelling of the tumour immune microenvironment (**Figure 1G**, **Supplementary Figures S1H, I**), with cell abundance calculations providing more intuitive visualization of these results (**Supplementary Figures S2C, D**). We calculated differentially expressed genes (DEGs) between the tumour and normal groups for each cell type (**Figure 1H**), with epithelial cells showing the most DEGs, followed by fibroblasts, indicating that these two cell types may undergo the most significant phenotypic changes during tumour progression. GO analysis of the upregulated DEGs revealed that myeloid cells were significantly enriched in cytokine production and apoptotic signalling pathways, whereas T cells were enriched mainly in NF-κB signalling and cytokine production-related pathways. Notably, multiple cell types respond to oxidative stress and hypoxia, particularly endothelial cells and fibroblasts, which are actively involved in angiogenesis-related pathways, reflecting the complex intercellular interaction network in the tumour microenvironment (**Figure 1I**).

### 3.2 Multiple cell types exhibit coordinated patterns of metabolic activation and matrix remodelling

**Figure 2:**
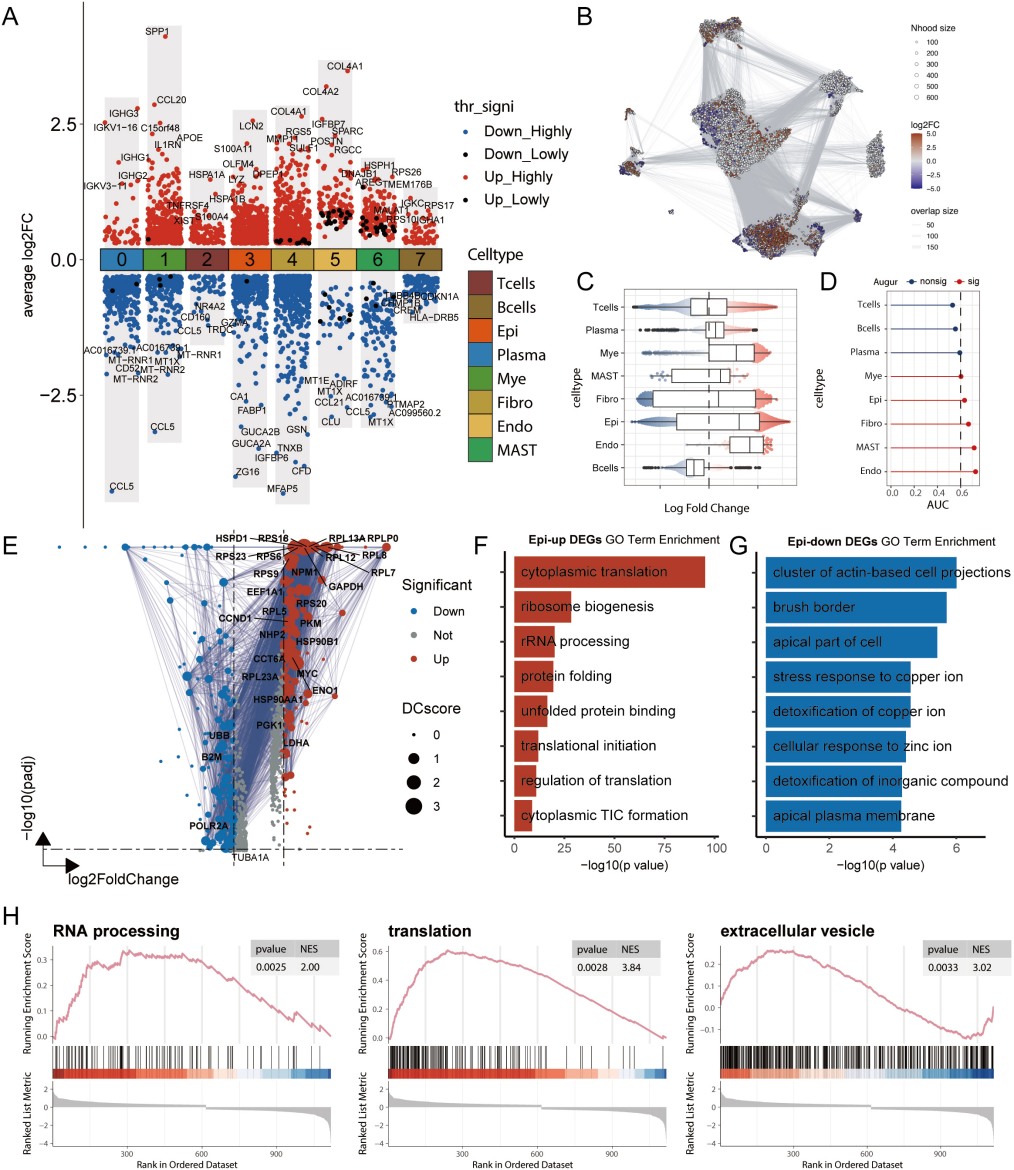
Multi-dimensional analysis reveals transcriptomic remodelling and functional changes in cell types in colorectal cancer. **(A)** Differentially expressed genes (DEGs) between tumour and normal groups across cell types, analysed using the Wilcoxon rank-sum test. Highly significant DEGs are defined as those with an adjusted p-value (adj.pval) < 0.05, while lowly significant DEGs are defined as those with adj.pval < 0.1. “Upregulated”refers to genes with higher expression in the tumour group relative to the normal group. **(B)** Differential abundance analysis using the Milo k-nearest neighbour (kNN) algorithm. Each node represents a local cellular neighbourhood, with colour intensity representing the log fold change of tumour relative to normal. White nodes indicate non-significant differences (FDR > 10%). The node layout is based on UMAP dimensionality reduction. **(C)** Statistical results of the Milo kNN differential abundance analysis. **(D)** Augur analysis framework assessing the degree of transcriptomic perturbation in each cell subtype between biological states. A higher AUC value indicates more significant transcriptomic alterations. **(E)** Volcano plot of DEGs in epithelial cells between tumour and normal groups. DC values represent protein-protein interaction network strength, calculated using the STRING database. **(F-G)** GO functional enrichment analysis of DEGs in epithelial cells. **(F)** Functional annotation of upregulated genes and **(G)** downregulated genes. **(H)** GSEA pathway enrichment analysis of DEGs in epithelial cells, showing representative signalling pathways. NES represents the Normalized Enrichment Score.

We performed a systematic analysis of transcriptional characteristics across cell types in tumour and normal tissues. First, through Wilcoxon rank-sum test analysis of DEGs, we identified changes in several key molecules: the chemokine *CCL5* was significantly downregulated in multiple immune cells (including T cells and myeloid cells), whereas *CCL20* was upregulated, suggesting a shift in the immune microenvironment from an antitumour status to a protumour status (27). Moreover, the concurrent upregulation of COL4A1/COL4A2 across multiple cell types, particularly in fibroblasts and endothelial cells, reflected extracellular matrix remodelling (**Figure 2A**). To comprehensively understand the changes in the cellular composition during disease states, we employed a multilayered analytical strategy. Differential abundance analysis based on k-nearest neighbour statistics (28) revealed significantly increased proportions of epithelial cells, endothelial cells, and myeloid cells in the tumour group (**Figures 2B, C**), suggesting expansion of the tumour parenchyma and active angiogenesis. Furthermore, using the random forest-based Augur algorithm (29) to assess transcriptional perturbation levels across cell types, we found that endothelial cells, mast cells, fibroblasts, and epithelial cells presented the most significant transcriptional changes (**Figure 2D**). Using AUCell, we calculated the pathway involvement of endothelial cells and mast cells in the tumour group and found that endothelial cells were involved primarily in extracellular matrix remodelling, whereas mast cells were involved mainly in lipid metabolism (**Supplementary Figures S2E, F**).

Given the dominant position of epithelial cells in samples and their significant abundance and transcriptional changes, we conducted in-depth functional analysis. GO enrichment analysis of the DEGs revealed that the upregulated genes were enriched mainly in protein synthesis- and ribosome biogenesis-related pathways, reflecting robust growth demands and stress adaptation responses of tumour cells, whereas the downregulated genes were enriched in cell polarity- and structure-related pathways, suggesting phenotypic alterations (**Figures 2E–G**). GSEA further confirmed the significant activation of three key pathways: RNA processing, translation, and extracellular vesicles. These changes not only reflect cancer cell proliferative activity and stress adaptation but also indicate tumour microenvironment remodelling and potential metastatic tendencies suggesting that systematic changes occur across multiple cell types in CRC (**Figure 2H**).

### 3.3 Epithelial cell heterogeneity and copy number variation-driven malignant progression in CRC

**Figure 3:**
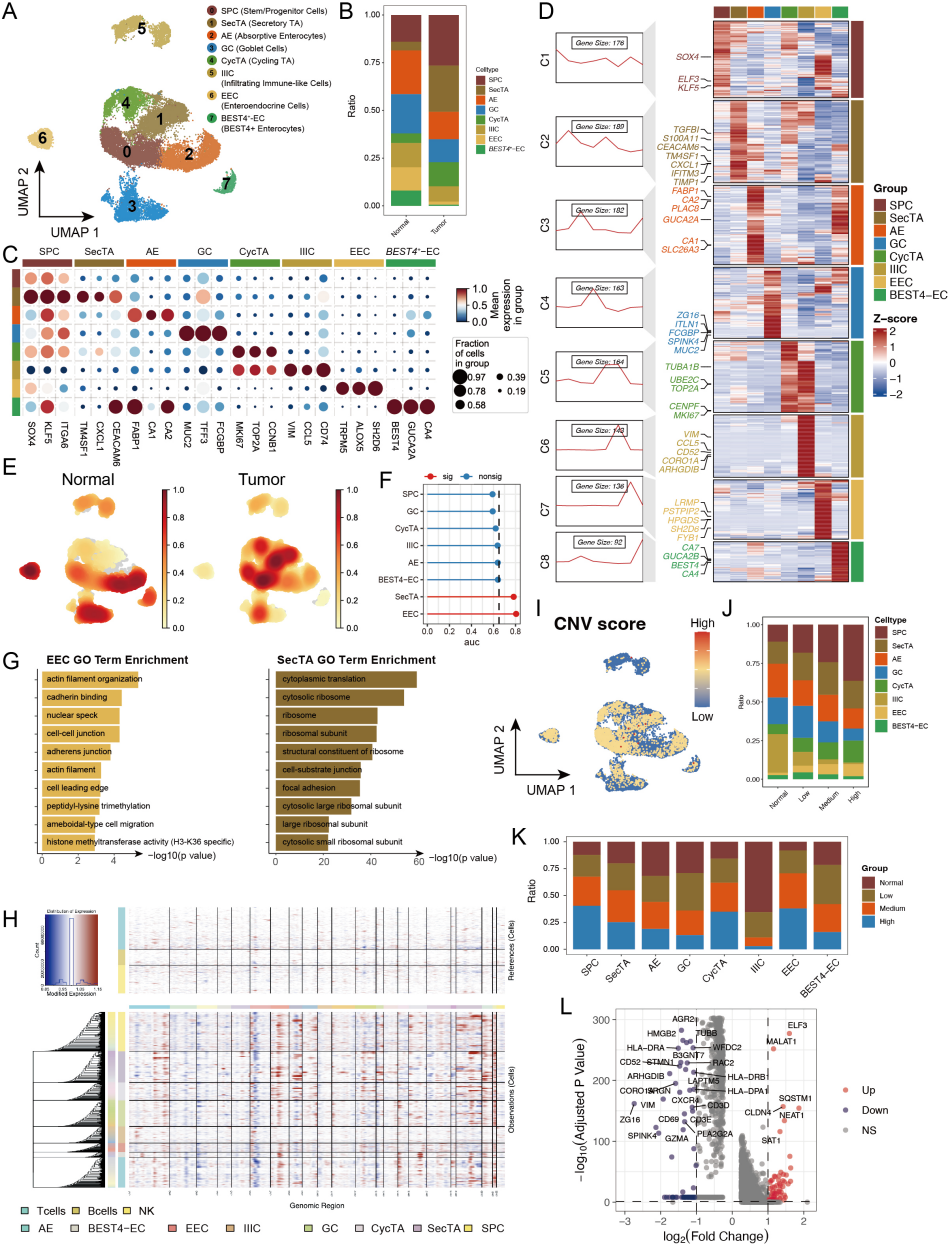
Copy number variation (CNV) analysis reveals genomic instability features in epithelial cell subtypes of colorectal cancer. **(A)** UMAP showing subtypes of stem/progenitor cells (SPCs), secretory transit amplifying cells (SecTA), absorptive enterocytes (AEs), goblet cells (GCs), cycling transit amplifying cells (CycTA), infiltrating immune-like cells (IIIC), enteroendocrine cells (EECs), and BEST4+ enterocytes (BEST4-ECs). **(B)** Stacked bar plot representing the proportional distribution of cell types across different groups. **(C)** Expression of marker genes used for the identification of each cluster. **(D)** Heatmap showing the average expression of genes in each epithelial cell subtype, with the left panel displaying clustering of subtype-specific genes and representative markers. **(E)** Density plot showing the enrichment of cell counts in each group. **(F)** Augur framework displaying transcriptomic perturbation across subclusters under two biological conditions, where a higher AUC represents greater transcriptomic changes. **(G)** GO enrichment analysis of differentially expressed genes (DEGs) in EEC and SecTA subtypes. **(H)** InferCNV analysis predicting copy number variations in each epithelial cell subtype using T cells, B cells, and NK cells as references. **(I)** CNV scores mapped onto the UMAP of epithelial cells, with colour intensity representing the CNV score. **(J)** Proportional distribution of cell types with different CNV statuses, identified based on CNV scores. **(K)** Proportion of cells with different CNV statuses within each cell type. **(L)** Volcano plot of DEGs between high CNV and normal cells.

Considering that epithelial cells are the primary source of malignant tumour cells in CRC and their significant perturbations at both the abundance and transcriptional levels, we conducted an in-depth analysis of epithelial cells. Through reclustering, we identified 8 distinct epithelial cell subgroups (**Figure 3A**, **Supplementary Figures S3A, B**), including stem/progenitor cells (SPCs), secretory transit amplifying cells (SecTA), absorptive enterocytes (AEs), goblet cells (GCs), cycling transit amplifying cells (CycTA), infiltrating immune-like cells (IIIC), enteroendocrine cells (EECs), and BEST4+ enterocytes (BEST4-ECs). On the basis of previous studies and analyses of highly expressed genes in each subgroup (30, 31), we validated these subgroup annotations (**Figures 3C, D**). By calculating subgroup-specific highly expressed genes, we not only confirmed cell identity specificity but also identified representative marker genes, such as the stem cell characteristic factors *SOX4, ELF3*, and *KLF5* in the SPCs and *VIM* and *CCL5* in infiltrating immune-like cells (**Figure 3D**). Additionally, we performed functional enrichment analysis on the genes that were specifically expressed in each subgroup to validate their functions (**Supplementary Figures S3C, D**). When comparing tumour and normal tissues, we detected significantly increased abundances of SPCs, SecTA, and CycTA in the tumour group, indicating the activation of stem cell properties and inflammatory responses, whereas decreased abundances of AEs, EECs, and BEST4-ECs reflected impaired normal absorption and endocrine functions, revealing significantly different epithelial cell states between the two groups (32, 33) (**Figures 3B, E**). Augur framework analysis revealed that EECs and SecTA were the two subgroups with the most significant transcriptional perturbations (**Figure 3F**). GO enrichment analysis of DEGs revealed that EEC-related genes were enriched mainly in cytoskeleton- and cell junction-related pathways, such as cell migration, cadherin binding, and cell–cell junctions, changes that might affect their paracrine regulatory function and hormone secretion polarity, potentially leading to invasive characteristics. SecTA DEGs were enriched mainly in protein synthesis and cell adhesion-related pathways, such as cytoplasmic translation and cytosolic ribosomes, reflecting significantly increased protein synthesis activity and metabolic levels in SecTA within tumour tissue, suggesting possible increased secretory protein production (**Figure 3G**).

To explore differences in malignancy among epithelial cell subtypes, we performed CNV analysis via the inferCNV tool (34) (**Supplementary Figure S3E**). The results revealed significant heterogeneity in CNV patterns among different epithelial cell subgroups, with IIIC showing the lowest CNV scores (**Figures 3H, I**, **Supplementary Figure S3F**). After the cells were categorized into four groups on the basis of their CNV levels (normal, low, medium, and high), we observed gradually increasing trends in SPCs, CycTA, and EECs with increasing CNV levels, which is consistent with existing research reports and reflects tumour cell malignancy (**Figure 3J**) (35, 36). When AUCell was used to evaluate activated pathways in epithelial cells with different CNV levels, high CNV significantly activated protein phosphorylation, transcription regulation, and DNA templating, suggesting increased active transcriptional and translational regulation (**Supplementary Figure S3G**). Notably, IIIC presented mainly normal and low CNV, whereas EECs and SPCs presented mainly medium or high CNV, suggesting that genomic instability might be acquired through enhanced stem cell properties and that CNV accumulation might be an important driving factor in the malignant transformation of these cells (**Figure 3K**). By comparing the DEGs between the high and normal CNV groups, we found that *MALAT1*, *ELF3*, and *CLDN4* were significantly upregulated in the high-CNV group, indicating significant changes in epithelial cell properties (such as EMT) and cell junction patterns (**Figure 3L**).

### 3.4 Pseudotime analysis reveals dynamic transformation trajectories of CRC epithelial cells

**Figure 4:**
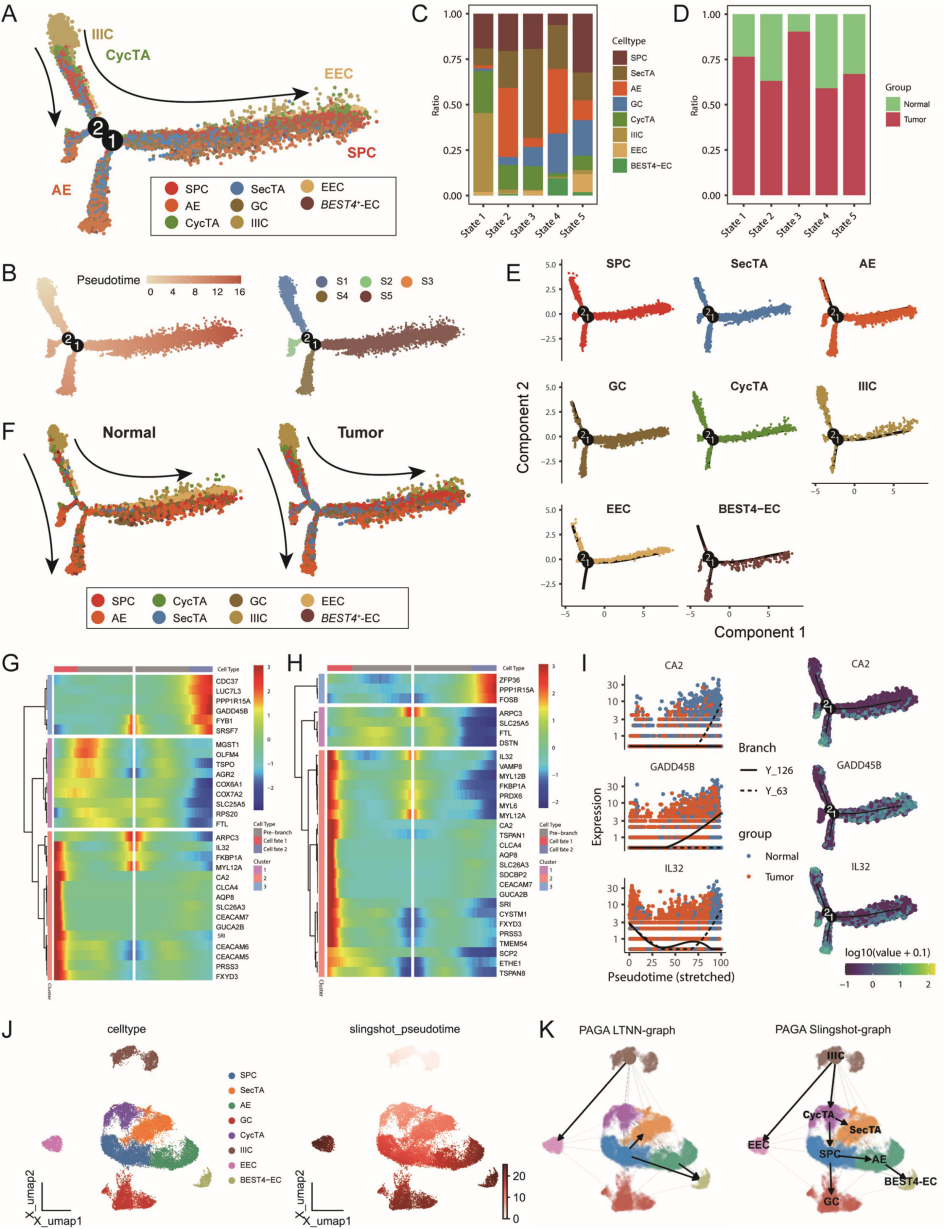
Multi-algorithm trajectory analysis reveals the dynamic transformation process of epithelial cells in colorectal cancer. **(A)** Differentiation trajectory of epithelial cell subtypes based on the Monocle algorithm, with different colours representing distinct cell types. **(B)** Developmental pseudotime and cell state distribution inferred from the Monocle analysis. **(C)** Proportional composition of different epithelial cell subtypes within each cell state. **(D)** Proportional composition of tumour and normal samples within each cell state. **(E)** Distribution trajectories of different epithelial cell subtypes along the pseudotime axis. **(F)** Comparative cell differentiation trajectories between tumour and normal groups, with cells coloured by subtype. **(G–H)** BEAM analysis identifying key transition node DEGs. Heatmaps displaying the top 30 genes with significant changes before and after **(G)** node 1 and **(H)** node 2. **(I)** Expression trends of representative genes CA2, GADD45B, and IL32 along the pseudotime axis, coloured by sample group and differentiation trajectory. **(J)** Cell differentiation trajectory inferred based on the Slingshot algorithm. **(K)** Left: Trajectory inference network constructed using the PAGA algorithm; Right: Integrated trajectory analysis results combining PAGA and Slingshot.

To analyse the dynamic transformation characteristics of different epithelial cell subtypes in the tumour and normal groups, we performed pseudotime analysis on 8 epithelial cell subtypes via the monocle package. On the basis of this analysis, we identified 5 distinct cell states (**Figures 4A, B**). By analysing the proportions of each cell type in each state, we found that in State1, approximately half of the cells were identified as IIICs, which is consistent with our previous CNV analysis finding that this cell type had the lowest CNV level. Additionally, AEs and SPCs were enriched at two different endpoints of the trajectory, whereas tumour group cells presented similar distribution patterns across all periods (**Figures 4C, D, F**). Further analysis of trajectory distribution characteristics for each cell type revealed that IIICs were enriched mainly at the starting point, EECs appeared primarily at the endpoint in the S5 direction, and BEST4-ECs were significantly enriched at both endpoints (**Figure 4E**). This distribution pattern suggests different differentiation paths that various cell types might undergo during tumour development.

To better understand the molecular mechanisms during cell state transitions, we studied gene expression changes before and after nodes 1 and 2 through BEAM analysis. As shown in **Figure 4G**, at node 1, Cluster 3 and Cluster 2 represented upregulated gene sets during development in the State5 and State4 directions, respectively. Cluster 3 was enriched with multiple cell stress-related genes, such as *PPP1R15A* and *GADD45B*; in particular, abnormal *GADD45B* expression has been reported to be closely related to CRC progression and prognosis (37, 38). Cluster2 was enriched with cytoskeleton and metabolism-related factors, such as *ARPC3* and *CA2*, where *CA2* participates in regulating the cellular pH and ion balance, and its expression changes suggest significant alterations in the tumour microenvironment (39). In the analysis around node 2, we observed enrichment of numerous immune-related genes at the starting point, such as *IL32* and *CEACAM7* (**Figures 4H, I**, **Supplementary Figures S4B, C**), indicating that immune regulation might play an important role in early cell state transitions and potentially participate in tumour microenvironment remodelling.

To validate the reliability of the trajectory inference, we simultaneously used two independent methods, SlingShot and PAGA, for analysis. Both methods identified IIICs as the trajectory starting point, which is highly consistent with the results of Monocle. Furthermore, they both identified BEST4-ECs and GCs as two endpoints of the trajectory, further supporting the view of multidirectional differentiation trajectories of epithelial cells during CRC progression and revealing the complexity and plasticity of epithelial cell fate determination during tumour development (40, 41) (**Figure 4J, K**, **Supplementary Figures S4D–F**). These findings not only help us understand the dynamic changes in CRC epithelial cells but also provide new perspectives for further studies of tumour progression mechanisms and the development of potential therapeutic strategies.

### 3.5 Immune cell heterogeneity and functional remodelling in the CRC microenvironment

**Figure 5:**
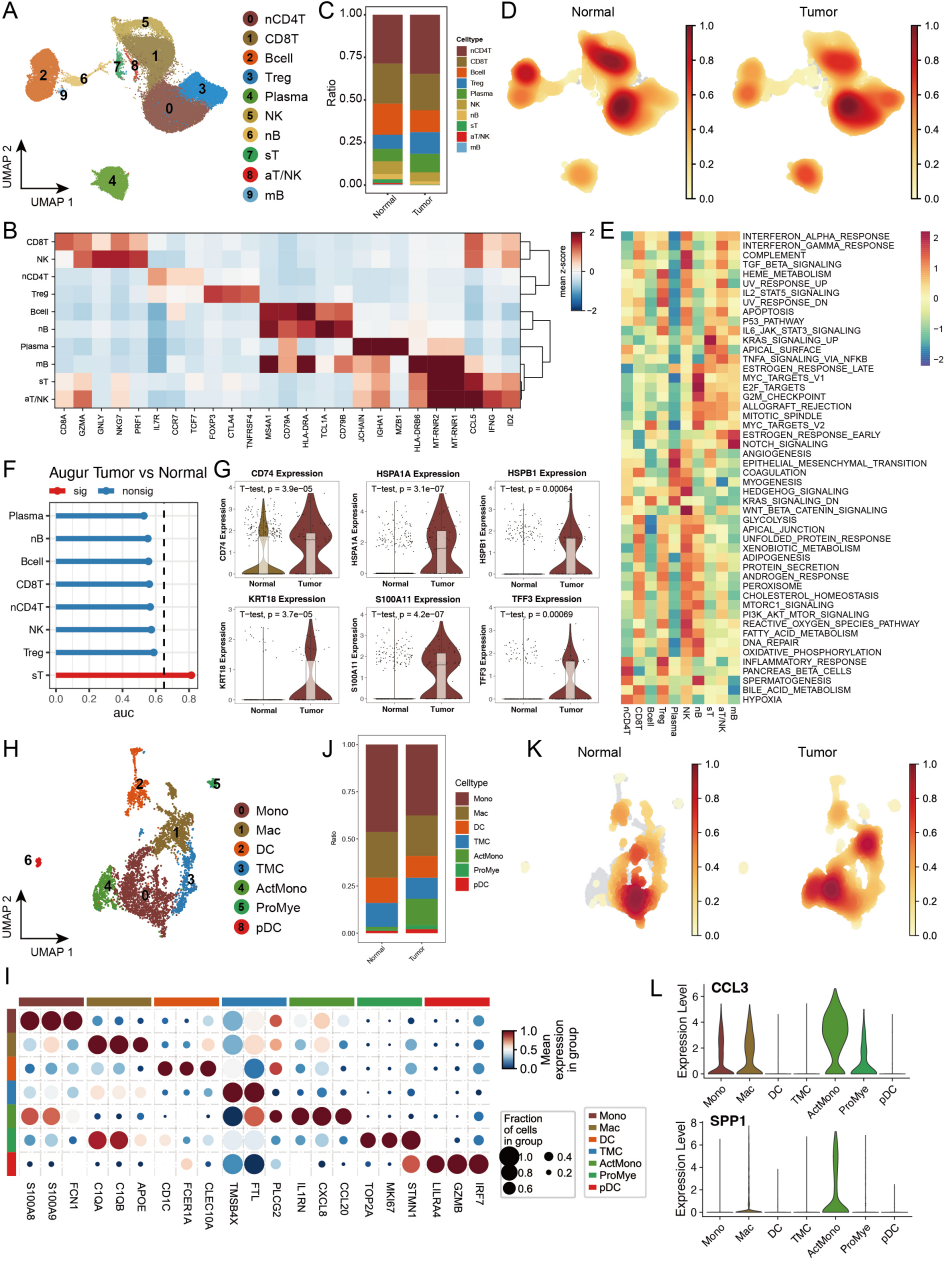
Single-cell transcriptomic analysis reveals the heterogeneity of lymphoid and myeloid cells in colorectal cancer. **(A)** UMAP dimensionality reduction plot depicting lymphoid cell subtypes, identifying ten major subtypes: Naive CD4+ T cells (nCD4T), Cytotoxic CD8+ T cells (CD8T), B cells (Bcell), Regulatory T cells (Treg), Plasma cells (Plasma), Natural Killer cells (NK), Naive B cells (nB), Stressed T cells (sT), Activated T/NK cells (aT/NK), and Memory B cells (mB). **(B)** Expression profiles of characteristic marker genes for each lymphoid cell subtype, used to confirm cell identity. **(C)** Stacked bar plot showing the proportional distribution of lymphoid cell subtypes in tumour and normal groups. **(D)** Density distribution plot of each lymphoid cell subtype in tumour and normal groups. **(E)** GSEA enrichment heatmap of 50 hallmark gene sets from the MSigDB database, demonstrating the functional characteristics of different lymphoid cell subtypes. **(F)** Transcriptomic perturbation assessment of each cell subtype under two biological conditions using the Augur framework, with AUC values reflecting the significance of transcriptomic changes. **(G)** Violin plots showing the expression levels of key marker genes. **(H)** UMAP dimensionality reduction plot depicting myeloid cell subtypes, identifying nine major subtypes: Monocytes (Mono), Macrophages (Mac), Dendritic Cells (DC), Transitional Myeloid Cells (TMC), Activated Monocytes/Macrophages (ActMono), Proliferating Myeloid Cells (ProMye), and Plasmacytoid Dendritic Cells (pDC). **(I)** Expression profiles of characteristic marker genes for each myeloid cell subtype. **(J)** Stacked bar plot showing the proportional distribution of myeloid cell subtypes in tumour and normal groups. **(K)** Density distribution plot of each myeloid cell subtype in tumour and normal groups. **(L)** Violin plots showing the expression levels of key marker genes in myeloid cells.

Considering our previous findings of significant perturbations in the immune response and immune microenvironment in the tumour group, we conducted an in-depth analysis of lymphoid and myeloid cells in our dataset. Through unsupervised clustering methods, we subdivided lymphoid cells into 10 distinct subgroups and annotated and confirmed their identities on the basis of subgroup-specific highly expressed genes (**Figure 5A, B**, **Supplementary Figures S5A–C**). Comparative analysis revealed that lymphoid cell subtypes in the tumour and normal groups presented similar overall distribution patterns, but nCD4T, Treg, and plasma cells presented slightly increased abundances in the tumour group, whereas B cells and NK cells presented significantly decreased abundances, suggesting enhanced immunosuppression and tumoural immune remodelling in the tumour microenvironment (**Figures 5C, D**).

To better understand the functional states of different immune cell subgroups, we evaluated pathway activation in various lymphoid cells via MSigDB characteristic gene sets. The results revealed unique pathway activation patterns across different lymphoid cells, with Treg cells significantly activating the IL2_STAT5_SIGNALING and IL6_JAK_STAT3_SIGNALING pathways, suggesting their important regulatory role in T-cell proliferation and differentiation. Regarding inflammation-related pathways, we observed that sT and aT/NK cells significantly activated the TNFα_SIGNALING_VIA_NFKB pathway, whereas INFLAMMATORY_RESPONSE was highly activated in nCD4T and Treg cells (**Figure 5E**). Through Augur framework analysis of transcriptional differences between the tumour and normal groups, we found that sT was the most significantly altered cell subgroup (**Figure 5F**). Further analysis revealed significant upregulation of multiple genes related to antigen stimulation and cellular stress in sT, including *CD74, HSPA1A*, *HSPB1*, and *S100A11*, suggesting that sT cells might be in multiple stress states, potentially affecting their normal immune function (**Figure 5G**).

In myeloid cell analysis, we identified 9 distinct cell subgroups and examined their functional marker expression characteristics in detail (**Figures 5H, I**, **Supplementary Figures S5D–F**). Cell abundance analysis revealed that while most myeloid cell subgroups maintained a relatively balanced distribution between the tumour and normal groups, ActMono was significantly upregulated in the tumour group (**Figures 5J, K**). Our pseudotime analysis revealed that both PAGA and SlingShot identified ActMono as an endpoint of myeloid cell differentiation (**Supplementary Figures S5G, H**), suggesting that ActMono might be a major cell subgroup responding in later stages of the cancer response. Additionally, the characteristic expression profile of this cell subgroup included multiple myeloid cell activation markers, such as *IL1RN, CXCL8*, *CCL20*, and the chemokines *CCL3* and *SPP1* (**Figure 5L**), indicating the activated state of myeloid cells in the tumour microenvironment.

### 3.6 Activation of multiple immune communications, including galectin, in the tumour group

**Figure 6:**
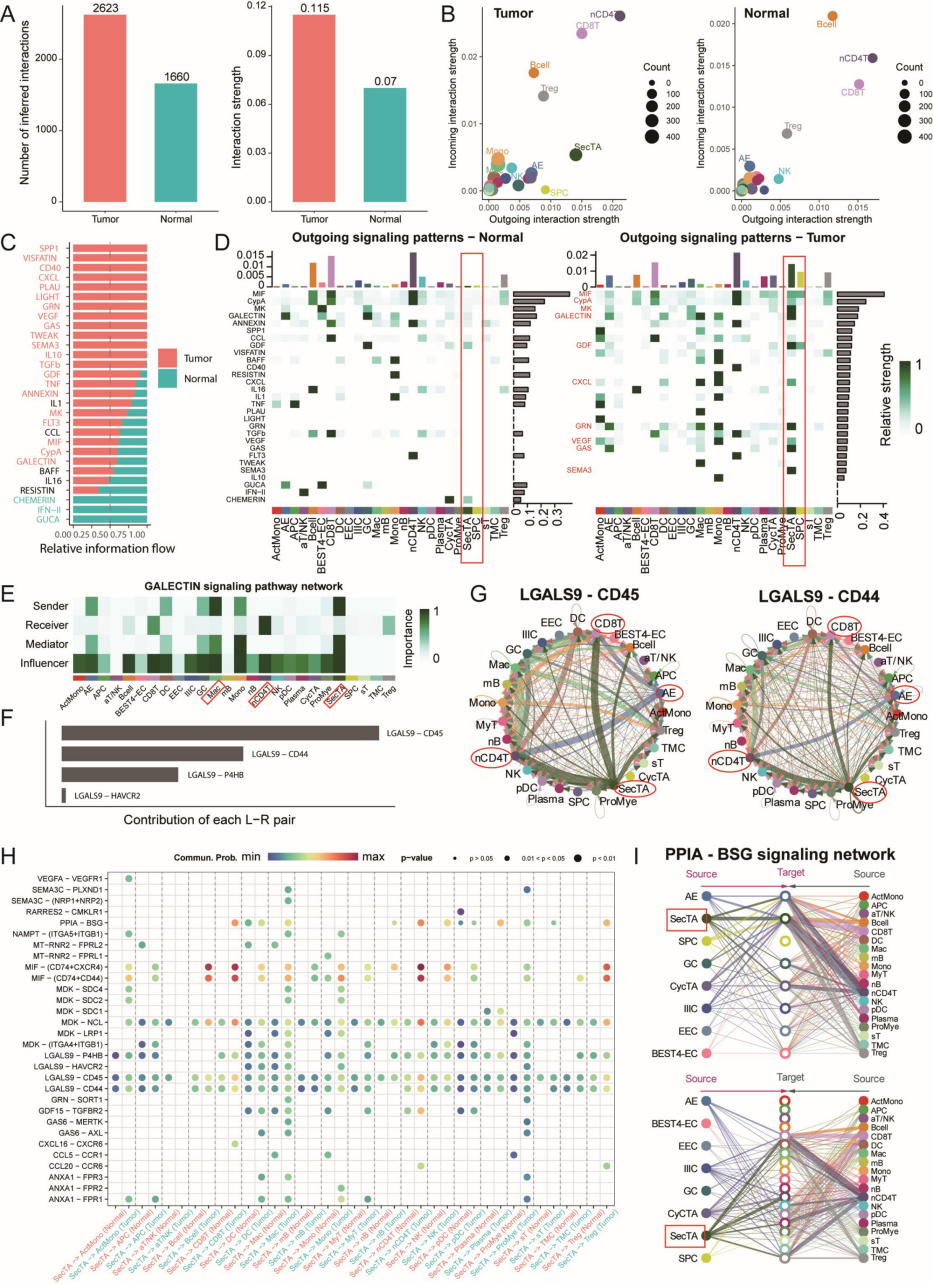
Cell communication network analysis reveals signal transduction remodelling in the colorectal cancer microenvironment. **(A)** Quantitative comparison of cell communication networks between tumour and normal groups, including the analysis of the number of signalling pathways and signal strength. **(B)** Scatter plot showing the incoming and outgoing signal strength of cell subtypes in tumour and normal groups, illustrating the roles of each subtype in the signalling network. **(C)** Comparative analysis of relative information flow for each signalling pathway between tumour and normal groups. **(D)** Heatmap displaying the outgoing signal strength of different cell types in tumour and normal groups. **(E)** Network localization and functional identification of cell types involved in the GALECTIN signalling pathway. **(F)** Contribution analysis of key receptor-ligand pairs in the GALECTIN signalling pathway. **(G)** Circular plot showing the involvement and signal strength of each cell type in specific receptor-ligand signalling pathways. **(H)** Bubble plot of receptor-ligand pairs specifically activated in the tumour group compared to the normal group, with SecTA as the signal source. Bubble size represents signal strength, and colour intensity indicates the significance of the difference. **(I)** Signal network analysis of cell types involved in the PPIA-BSG signalling pathway.

In the tumour microenvironment, cell–cell interactions play crucial roles in disease progression. On the basis of our previous identification of CRC epithelial and immune cell subgroups, we used the CellChat analysis platform to explore intercellular communication networks during disease progression in detail. The analysis results revealed that the cancer group presented greater communication numbers and signal intensities than the normal group did (**Figure 6A**, **Supplementary Figure S6A**). Signal transmission patterns were significantly different between the tumour and normal groups; in the tumour group, nCD4T cells were the main signal sending centre, followed by CD8T cells; however, in the normal group, B cells were the primary signal receivers. Compared with those in the normal group, SecTA and SPCs in the tumour group presented stronger signal sending capabilities, indicating significant functional remodelling of epithelial cell subgroups in the tumour state (**Figure 6B**). Further analysis revealed cancer group-specific activation of several important signalling pathways, including the CD40 pathway, which regulates immune response intensity; the SPP1 pathway, which mediates cell adhesion and microenvironment remodelling; and key signalling pathways, such as the VEGF and TGFβ pathways (**Figure 6C**).

We focused particularly on the signal characteristics emitted by SecTA and SPCs in tumours. As shown in **Figure 6D**, SPCs emitted mainly MIF and GDF signals, whereas SecTA presented a more complex signal network, including growth factor signals (*MK, GDF*), chemokine signals (CXCL), immune regulatory signals (galectin), and metabolic regulatory signals (GAS). This diverse signalling pattern suggests that SecTA might play important roles in tumour microenvironment remodelling, especially in immune cell recruitment and matrix reconstruction. Moreover, we observed AE signal activation, mainly manifested as GRN and GAS signal transmission, reflecting epithelial cell functional differentiation during tumour progression. Regarding signal reception in the tumour group, as previously predicted, SecTA showed a certain signal intensity, whereas SPCs showed no signals; overall, SecTA and SPCs played major communication roles compared with the other epithelial cell types (**Supplementary Figures S6B, C**).

Particularly noteworthy is the galectin signalling pathway, which, according to previous studies, participates in various immune responses and affects T-cell function, potentially promoting tumour immune escape and the formation of an immunosuppressive microenvironment (42, 43). Pathway analysis revealed that SecTA and Mac cells were the main signal emitters, whereas nCD4T cells were the main receivers, with this process being regulated by multiple cell types (**Figure 6E**). Among these, LGALS9-CD45/CD44 was the receptor–ligand pair with the greatest contribution (**Figure 6F**). In this signalling network, SecTA and AEs transmit signals to CD8T and nCD4T cells, with nCD4T cells subsequently transmitting signals to DC, forming a complex signal cascade network (**Figure 6G**). Comparative analysis revealed specific receptor–ligand interaction patterns between tumour group SecTA, SPCs, and immune cells. In addition to the galectin pathway, SecTA interact with various immune cells through the MIF-(CD74+CXCR4/CD44) signalling network, suggesting its important role in myeloid cell recruitment, activation, and T-cell activation (**Figure 6H**). Additionally, we examined signals transmitted from immune cells to SecTA and found that PPIA-BSG signalling appeared to be specific and exclusively interactive with SecTA (**Supplementary Figure S6D**), with the crucial role of this pathway in various diseases previously reported (44). The diversity of the incoming and outgoing patterns of epithelial cells was also predicted (**Supplementary Figures S6E, F**). Overall, the tumour group presented more complex receptor–ligand interaction networks, revealing specific changes in cellular communication within the tumour microenvironment.

### 3.7 SecTA subgroup-related features can predict patient survival

**Figure 7:**
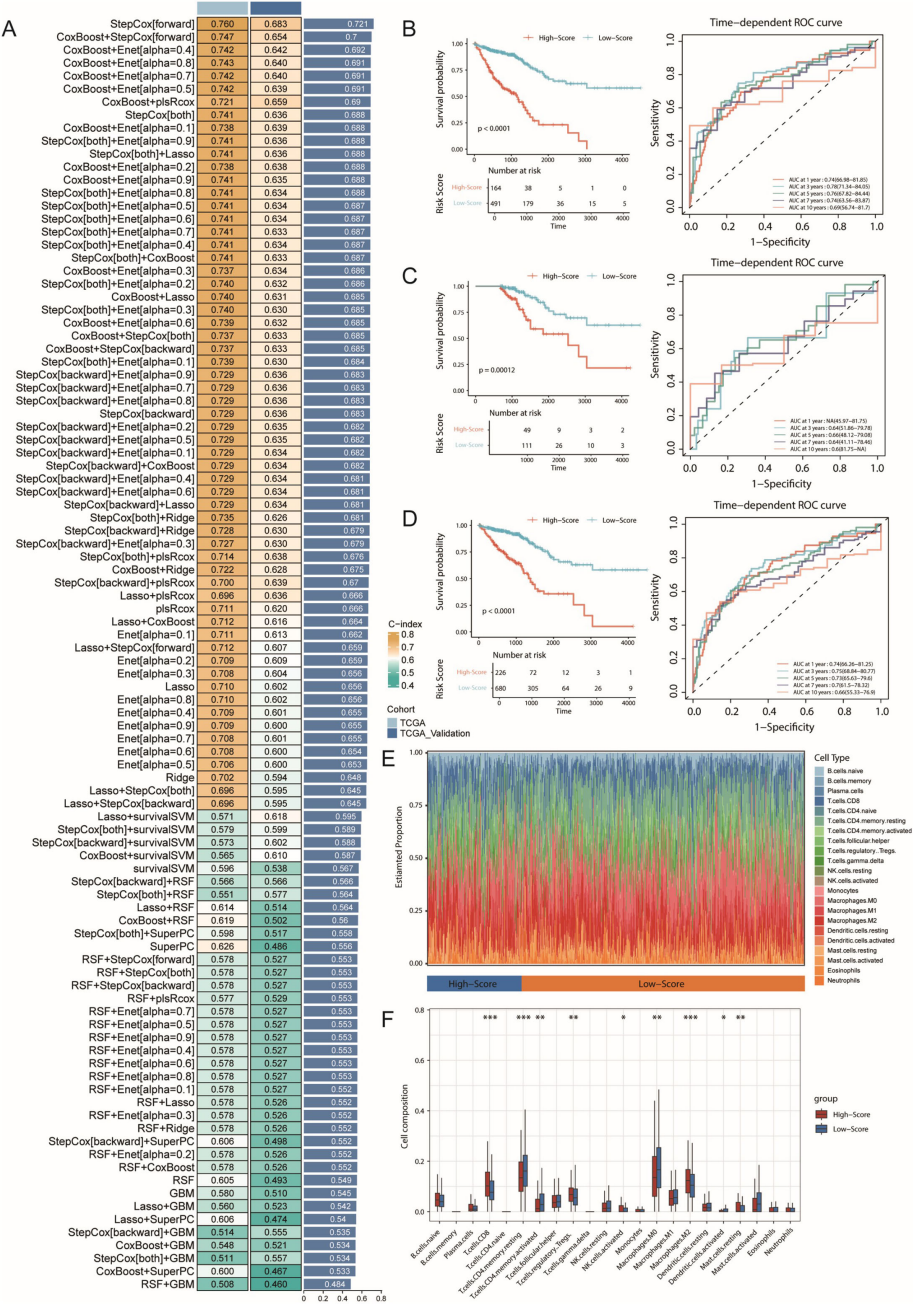
Machine learning-based prognostic model construction for colorectal cancer and its immunological characteristics analysis. **(A)** Construction and validation of the consensus feature signature CRS using a machine learning-based ensemble approach. A total of 101 predictive models were generated using a LOOCV framework, and the C-index for each model was calculated across all training and validation datasets. **(B)** Kaplan-Meier survival curve and ROC curve of the CRS prognostic model in the training set, with 1-year, 3-year, and 5-year AUC values used to evaluate the model’s performance. **(C)** Kaplan-Meier survival curve and ROC curve of the CRS prognostic model in the validation set. **(D)** Kaplan-Meier survival curve and ROC curve of the CRS prognostic model in all samples. **(E)** Immune infiltration analysis of high- and low-risk groups identified by the model, using the CIBERSORT method. **(F)** Boxplot showing the infiltration levels of various immune cell types. * represents p < 0.05, ** represents p < 0.01, *** represents p < 0.001.

To evaluate the molecular characteristics associated with CRC prognosis, we integrated the TCGA-COAD and READ datasets with their prognostic information to construct prediction models through multidimensional gene expression analysis. First, we performed intersection analysis of SecTA-specific DEGs with high CNV group DEGs and tumour-normal epithelial cell DEGs, resulting in the identification of 282 candidate genes. To establish a robust prediction model, we employed a leave-one-out cross-validation (LOOCV) framework, constructing and evaluating 101 prediction models. We randomly divided the TCGA dataset into training and validation sets at a 6:4 ratio and evaluated model performance through the C-index. The results showed that while the StepCox[forward] model achieved the highest average C-index (0.721), it required 80 features for survival fitting. In contrast, the StepCox[both]+Enet[alpha=0.7] model achieved an average C-index of 0.687 when only 25 features were used, demonstrating better practicality (**Figure 7A**). On this basis, we selected the StepCox[both]+Enet[alpha=0.7] strategy to construct the CRC risk score (CRS) system.

The CRS model demonstrated good predictive performance in both the training and validation sets. In both datasets, the low-risk group had significantly prolonged overall survival (OS, p<0.0001; **Figure 7B**). With respect to prediction accuracy, in the training set, the CRS achieved AUCs of 0.74 (95% CI: 66.98**–**81.85), 0.78 (95% CI: 71.34**–**84.05), and 0.76 (95% CI: 67.82**–**84.44) for 1-year, 3-year, and 5-year OS prediction, respectively. Although the validation set could not calculate the 1-year AUC due to sample size limitations, the AUC values for 3-year and 5-year OS still reached 0.64 (95% CI: 51.86–79.78) and 0.66 (95% CI: 48.12–79.08), validating the model’s predictive value (**Figure 7C**). Analysis of the combined training and validation sets further confirmed the predictive ability of the CRS; the low-risk group maintained significantly prolonged survival in the overall sample (**Figure 7D**), with AUCs of 0.74 (95% CI: 66.26–81.25), 0.75 (95% CI: 68.84–80.77), and 0.73 (95% CI: 65.63–79.6) for 1-year, 3-year, and 5-year OS, respectively.

To better understand the associations between CRS and the tumour immune microenvironment, we analysed immune cell infiltration characteristics in the high- and low-risk groups via the CIBERSORT algorithm (**Figures 7E, F**). The results revealed significantly increased infiltration proportions of CD8+ T cells and M2-type macrophages (p<0.001) in the high-risk group, whereas the proportions of resting CD4+ memory T cells were significantly decreased (p<0.001). These changes in immune cell composition suggest the possible existence of a stronger immunosuppressive microenvironment in the high-risk group, characterized by increased numbers of M2-type macrophages, indicating the formation of an immunosuppressive microenvironment, potentially suppressed CD8+ T-cell function, and possibly weakened effector T-cell responses. These findings are somewhat consistent with our previous single-cell analysis results.

### 3.8 *TUBB* participates in CRC progression through regulation of the extracellular matrix remodelling

**Figure 8:**
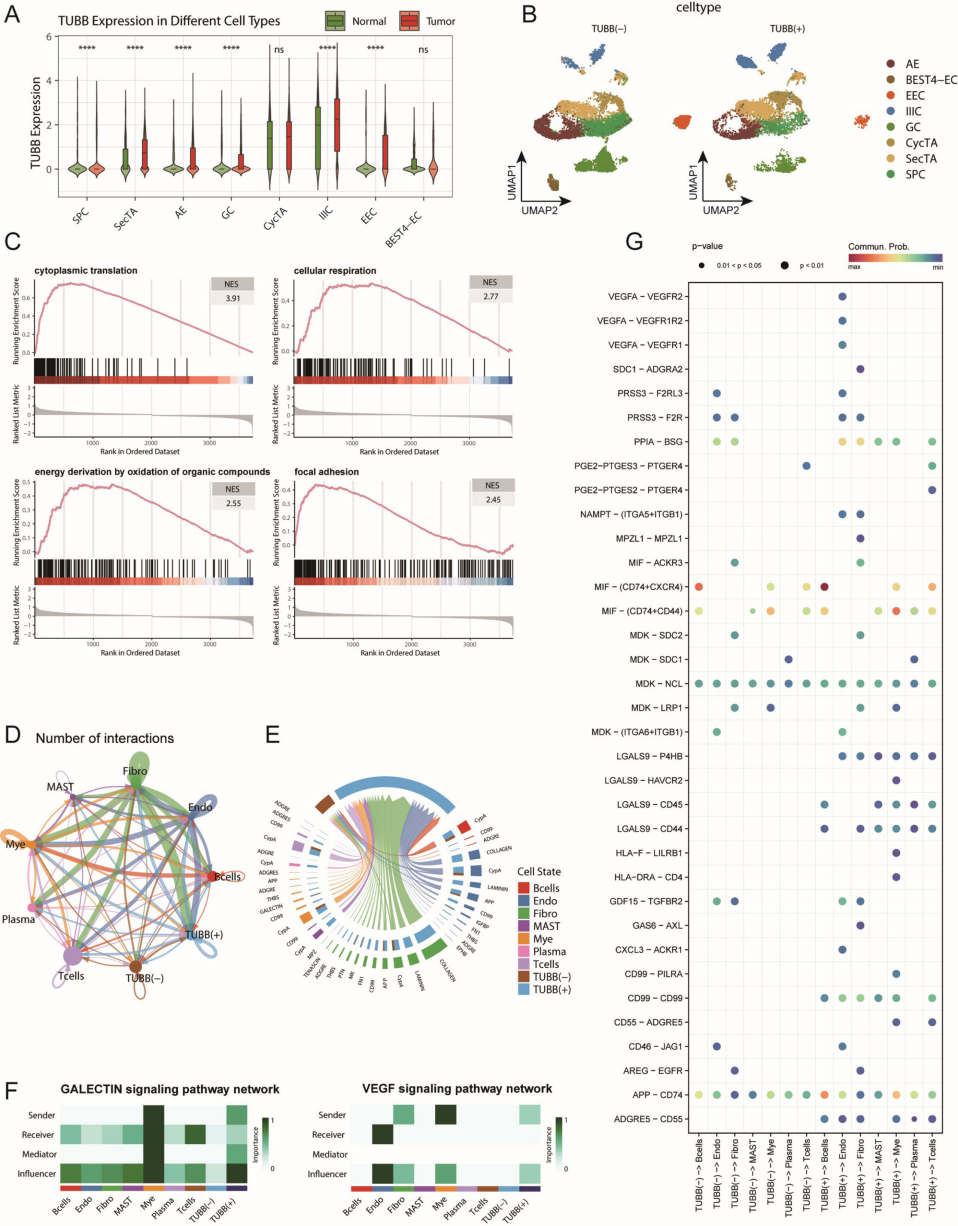
TUBB expression characteristics and its functional analysis in the colorectal cancer cell communication network. **(A)** Distribution of TUBB expression across different epithelial cell subtypes, showing expression levels in both tumour and normal groups. ns: not significant, ****p < 0.001 **(B)** t-SNE dimensionality reduction plot of epithelial cells based on TUBB expression levels, dividing the cells into TUBB-positive [TUBB(+)] and TUBBnegative [TUBB (–)] groups. **(C)** GSEA enrichment analysis showing representative signalling pathways associated with TUBB expression. **(D)** Circular plot displaying the interaction network strength between TUBB-positive/negative epithelial cells and various immune cells. **(E)** Analysis of the main signalling pathways received by TUBB-positive and TUBB-negative cell groups as signal recipients. **(F)** Heatmap shows the scores of each cell type involved in the GALECTIN and VEGF signalling pathways, including their roles as Senders, Receivers, Mediators, and Influencers. **(G)** Bubble plot showing the interaction patterns between TUBB-positive and TUBB-negative epithelial cells as signal senders and immune cells. Bubble size represents interaction strength, while colour intensity indicates significance.

Among the 25 feature genes in CRS, *TUBB* attracted our special attention. Previous studies have revealed the role of *TUBB* as a prognostic marker in pan-cancer (45), including breast cancer (46), and its carcinogenic role with miR-195 in lung cancer (47), but its role in CRC remains unclear. This prompted us to investigate the potential role of *TUBB* in CRC progression in detail. First, we analysed the expression patterns of *TUBB* across different epithelial cell subtypes in both normal and tumour tissues. The results revealed elevated *TUBB* expression levels in most tumour epithelial cells, with particularly significant upregulation in EEC and IIICs (**Figure 8A**). This change in expression pattern suggests that *TUBB* might be associated with the malignancy of colorectal cancer epithelial cells. Furthermore, we classified epithelial cells based on *TUBB* expression levels. Among the cells expressing *TUBB*, their distribution showed no obvious cell type specificity (**Figure 8B**, **Supplementary Figure S7A**). This expression pattern suggests that *TUBB* might mark a special cellular state rather than a specific cell subtype. To understand the functional significance of *TUBB*, we performed differential gene analysis and GSEA enrichment analysis between *TUBB*-positive and *TUBB*-negative cells. The results showed significant upregulation of pathways related to cytoplasmic translation, cellular respiration, energy derivation by oxidation of organic compounds, and other related pathways, including focal adhesion, in *TUBB*-positive cells. These characteristics are consistent with typical pro-cancer phenotypes, suggesting that *TUBB* may have oncogenic effects (**Figure 8C**).

Using the CellChat tool to analyse interactions between *TUBB*-positive and *TUBB*-negative epithelial cells and immune cells (**Figure 8D**, **Supplementary Figure S7B**), we identified two key features: First, *TUBB*-positive cells significantly received COLLAGEN, LAMININ, and VEGF signals. Notably, the activation of GALECTIN and VEGF signalling suggests that *TUBB*-positive cells possess immunosuppressive and pro-angiogenic capabilities, indicating their potential role in tumour progression and metastasis (**Figures 8E-F**, **Supplementary Figure S7C**). These extracellular matrix component signals are typically dominated by fibroblasts and endothelial cells, and the participation of TUBB-positive epithelial cells in such signalling suggests their potential role in microenvironmental remodelling. Specific receptor-ligand pairs in *TUBB*-positive cells include LGALS9-P4HB/CD44/CD45, among others (**Figure 8G**, **Supplementary Figures S7D-E**). This matrix remodelling may affect tumour progression by altering tissue stiffness and matrix density (48, 49). Additionally, both groups predominantly sent signals such as MHC-I, MIF, and APP (**Supplementary Figure S7B**). Second, we found that *TUBB*-positive cells also participated in the transmission of multiple specific signals, including CD99 and HLA-F (**Figure 8G**, **Supplementary Figure S7C**). The involvement in these signalling pathways suggests that *TUBB* may influence disease progression by regulating immune responses. These findings collectively depict the complex functions of *TUBB* in CRC: on one hand, it may promote tumorigenesis by enhancing cell migration, while on the other hand, it may influence disease progression by regulating the microenvironment and immune responses.

### 3.9 The clinical exploration and validation of *TUBB*

**Figure 9:**
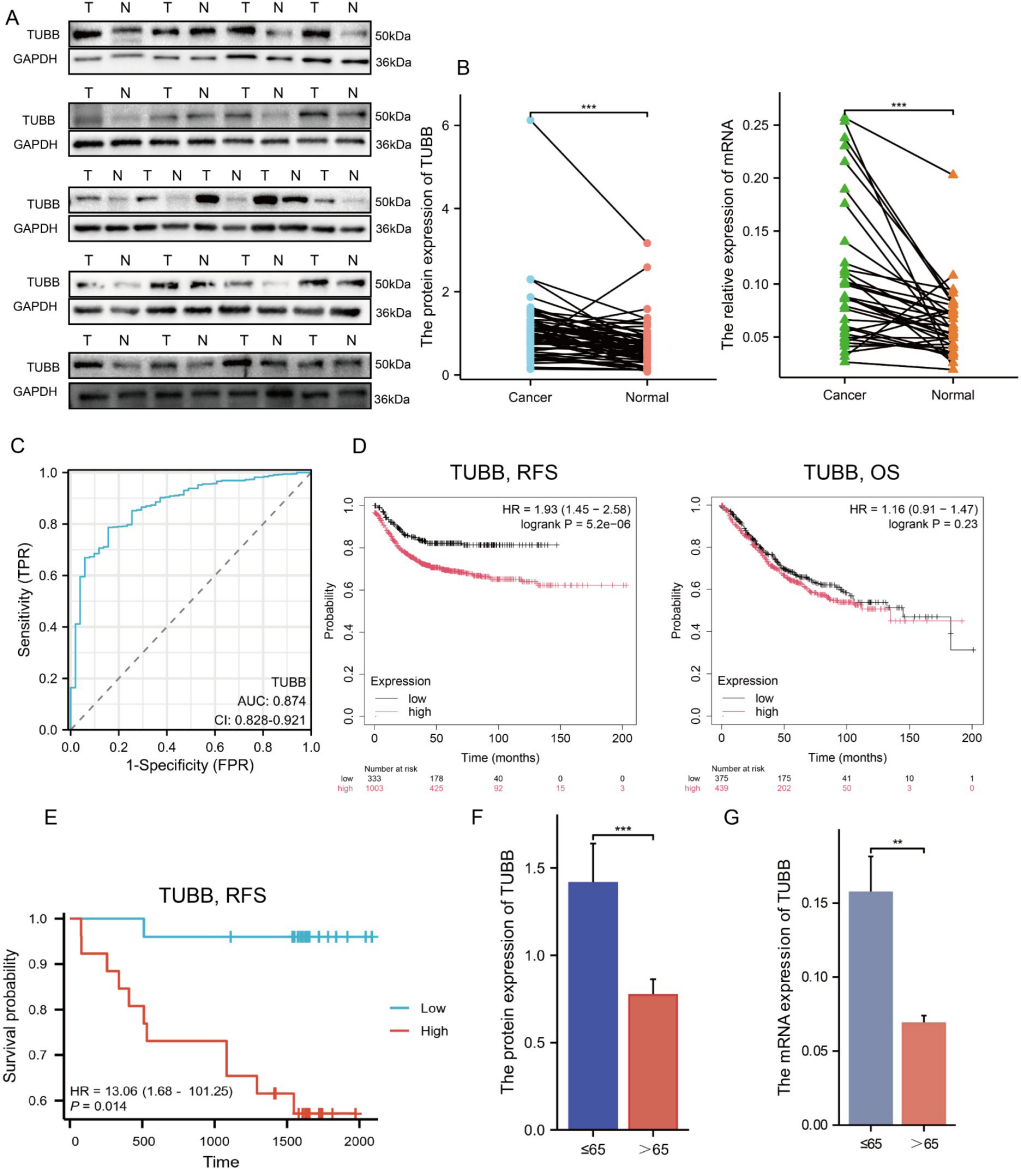
Exploration and confirmation of the clinical significance of TUBB. **(A)** The protein expression of TUBB in 21 pairs of tissues. **(B)** The mRNA expression of TUBB in the enrolled 35 patients. **(C)** Receiver operating characteristic curve for TUBB expression in normal samples and adjoining CRC tissues and samples from TCGA. **(D)** RFS and OS were expressed using Kaplan–Meier survival curves. **(E)** Kaplan–Meier survival curves of RFS in cohort from Yangpu Hospital (n=59). **(F, G)** Differences in TUBB expression in age subgroups at protein and mRNA level (n=59). OS, overall survival; RFS, recurrence-free survival. **P < 0.01, ***P < 0.001 compared to the corresponding groups.

To further investigate the clinical significance of *TUBB*, we obtained paired clinical tissue samples from colorectal cancer patients at Yangpu Hospital, utilizing these for Western Blot (n=59) and quantitative Reverse Transcription Polymerase Chain Reaction (qRT-PCR) analyses (n=35). The results from Western Blot indicated a notable increase in the expression levels of the *TUBB* gene in cancerous tissues compared to adjacent non-cancerous tissues (**Figure 9A**, **Supplementary Figure S8**), a result that was corroborated by qRT-PCR at the mRNA level (**Figure 9B**). Following these observations, we performed diagnostic Receiver Operating Characteristic (ROC) analyses (**Figure 9C**) and KM Plotter evaluations (**Figure 9D**) on *TUBB*, further validating its diagnostic and prognostic predictive capabilities through survival analysis of our colorectal cancer cohort (n=59) (**Figure 9E**). Additionally, we accessed clinical data and gene expression profiles of 644 colorectal cancer cases from the TCGA database for this analysis (**Table 1**). Our findings revealed a significant correlation between *TUBB* expression levels and variables such as age and CEA levels. By analysing the differential expression of *TUBB* at the protein and mRNA levels in clinical samples from our hospital, we have confirmed the difference *TUBB* expression between different age groups (**Figures 9F, G**). Consequently, our research emphasizes the association of *TUBB* with clinical characteristics of CRC.

**Table 1:** Clinical characteristics of patients with colorectal cancer.

Characteristics	Low expression of TUBB	High expression of TUBB	P value
n	322	322	
Pathologic T stage, n (%)			0.411
T1&T2	61 (9.5%)	70 (10.9%)	
T3&T4	258 (40.2%)	252 (39.3%)	
Pathologic N stage, n (%)			0.873
N0	183 (28.6%)	185 (28.9%)	
N1&N2	137 (21.4%)	135 (21.1%)	
Pathologic M stage, n (%)			0.195
M0	226 (40.1%)	249 (44.1%)	
M1	49 (8.7%)	40 (7.1%)	
Pathologic stage, n (%)			0.956
Stage I&Stage II	175 (28.1%)	174 (27.9%)	
Stage III&Stage IV	138 (22.2%)	136 (21.8%)	
Age, n (%)			0.011
<= 65	122 (18.9%)	154 (23.9%)	
> 65	200 (31.1%)	168 (26.1%)	
Gender, n (%)			0.693
Female	148 (23%)	153 (23.8%)	
Male	174 (27%)	169 (26.2%)	
CEA level, n (%)			0.049
<= 5	123 (29.6%)	138 (33.3%)	
> 5	88 (21.2%)	66 (15.9%)	

### 3.10 *TUBB* promotes the viability, anti-apoptosis and metastasis

**Figure 10:**
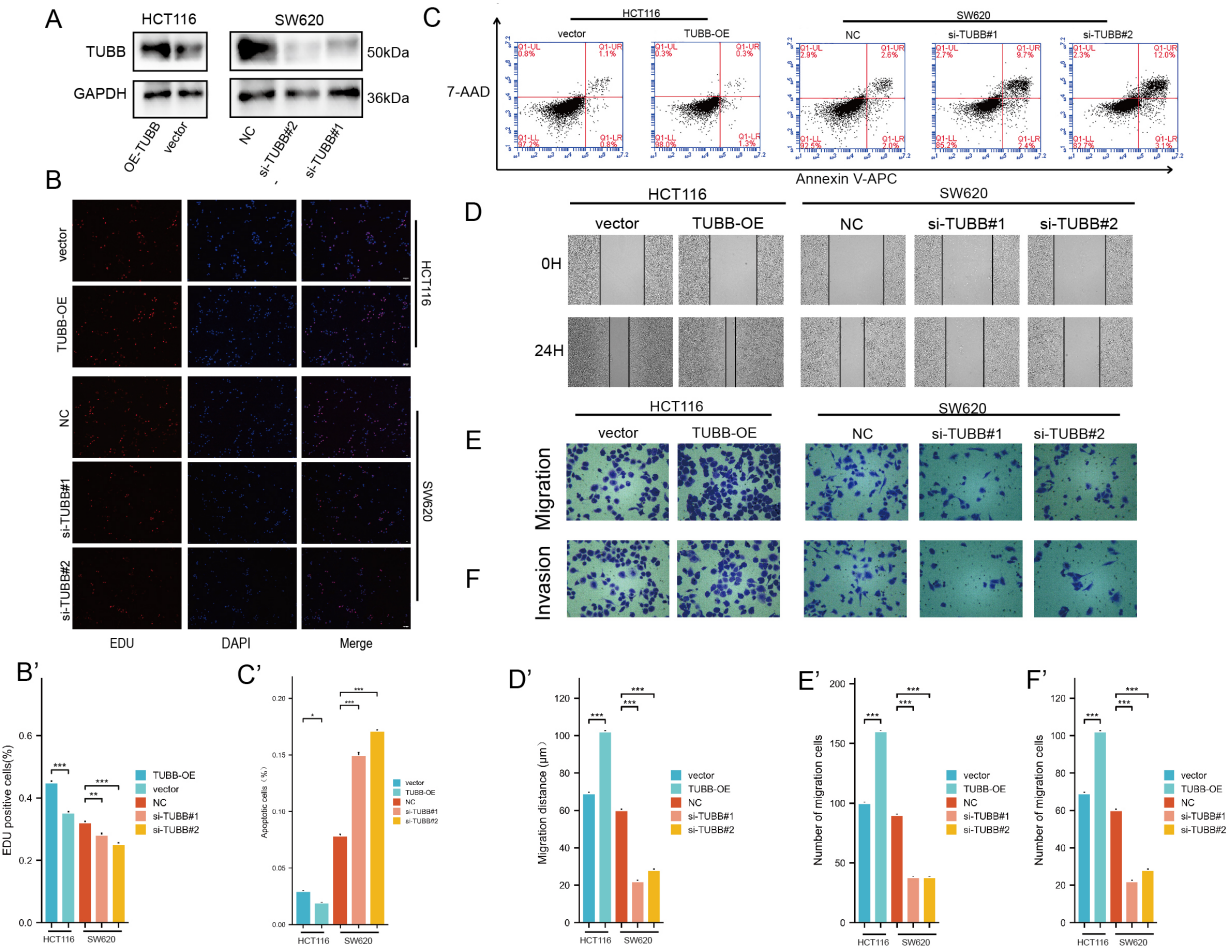
**(A)** Protein expression levels of TUBB were detected by western blotting. **(B)** EDU assay analysis. **(C)** Apoptosis rate was detected by flow cytometry. **(D-F)** Migration and invasion assay analysis. *P < 0.05, **P < 0.01, ***P < 0.001 compared to the corresponding groups.

In order to explore the functions of *TUBB* in CRC, *TUBB* was knocked down by siRNA in SW620 and overexpressed in HCT116, and the efficiency was verified by western blotting (**Figure 10A**).

EDU results showed that SW620 had a decreased proliferation, while HCT116 had an increased viability (**Figure 10B**). Furthermore, we detected the effect of *TUBB* on CRC apoptosis, and our study indicated that overexpression of *TUBB* significantly reduced the apoptosis rate of CRC cells, while knockdown of *TUBB* significantly increased the apoptosis rate of CRC cells (**Figure 10C**). The wound healing assay showed a marked decrease in cell migration following *TUBB* knockdown (**Figure 10D**) and an increase after its overexpression. Consistent with the results, transwell assays verified that *TUBB* knockdown inhibited SW620 invasion and migration, and its overexpression in HCT116 had the opposite trend (**Figures 10E, F**).

## 4 Discussion

CRC is a malignant tumour with high global incidence and mortality rates and is characterized by significant molecular heterogeneity and a complex tumour microenvironment (50, 51). Although traditional treatments such as surgery, chemotherapy, and targeted therapy have made some progress, the prognosis for advanced patients remains poor. While current single-cell sequencing studies have revealed heterogeneous characteristics of CRC cell populations and their interactions with the microenvironment, providing new perspectives for understanding tumour progression mechanisms and immune escape, individual datasets are often limited by sample size and population characteristics, making it difficult to comprehensively capture disease heterogeneity and complexity (10, 52). Therefore, integrating multiple CRC single-cell datasets is particularly necessary. In this study, we analysed 41 samples from 4 datasets, revealing not only the cellular diversity in stromal and immune components but also the identification of multiple cell subgroups that may play key roles in disease development, such as the SecTA subgroup with special proliferation and invasion characteristics. More importantly, our study highlights the complexity of cellular crosstalk in CRC, which may be a key factor driving tumour microenvironment remodelling. These findings provide certain clues for the development of new therapeutic strategies.

Among epithelial cell subtypes, we found EECs and SecTA to be significantly perturbed subgroups in the tumour group, and in our cell communication analysis, we emphasized the important roles of SecTA and SPCs in communication networks. We used AUCell to score each epithelial cell subtype and calculated significantly activated pathways, distinguishing them from other subtypes. For SecTA, positive regulation of cell population proliferation, positive regulation of cellular processes, and regulation of apoptotic processes were three specifically activated pathways, suggesting a highly active state (**Supplementary Figure S4A**). Combined with its highly expressed genes, we speculate that SecTA play a multifunctional promoter role in CRC progression. By maintaining high proliferative activity and resisting apoptotic signals, SecTA have significant survival advantages; moreover, these cells actively participate in extracellular matrix remodelling by secreting factors such as *TGFBI* and *TIMP1* and combining with CXCL1-mediated angiogenesis and immune cell recruitment, effectively reconstructing the tumour microenvironment (53–55). More importantly, SecTA expresses multiple molecules related to cell migration and adhesion, such as *CEACAM6* and *TM4SF1*, which not only increase tumour cell invasiveness but also create conditions for distant metastasis (56, 57). Additionally, by regulating inflammatory responses and immune cell recruitment, SecTA may participate in shaping an immune microenvironment favourable for tumour growth. This complex regulatory network makes it a key cell subgroup driving tumour progression, which is also verified in the cell communication section.

We found that sT cells were the most significantly perturbed lymphoid cell subgroup between the tumour and normal groups. This subgroup was highly expressed not only in mitochondrial genes but also in ribosome-related genes and multiple cell stress-related genes. This suggests both high cellular metabolic levels and high energy demands on the one hand and active protein synthesis on the other hand, indicating that this T-cell subgroup is in a stress state (58, 59). When we analysed myeloid cell heterogeneity, the ActMono (activated monocytes) state was significantly enriched in tumour tissues. Through pseudotime analysis, multiple algorithms consistently identified this subgroup as one of the main endpoints of myeloid cell differentiation, suggesting the reprogramming effect of the tumour microenvironment on immune cell fate determination. To better understand the functional characteristics of ActMono, we used the AUCell algorithm for pathway enrichment analysis of various myeloid cells (**Supplementary Figures S5I, J**). The results revealed that ActMono activated a series of characteristic pathways: first, the highly activated state was maintained through the activation of metabolic pathways such as protein folding and NAD biosynthesis; second, these cells maintained innate immune functions such as antimicrobial peptide production and Toll-like receptor signal transduction (60, 61); and, most importantly, they showed the ability to regulate T-cell proliferation and migration, suggesting a potential pivotal role in connecting innate and adaptive immune responses. This combination of functional characteristics makes ActMono a potential microenvironment regulator (62). On the one hand, its accumulation in tumour tissue might reflect changes in the immune microenvironment; on the other hand, through its unique immunomodulatory functions, it might influence the shaping of the microenvironment. This bidirectional effect might not only influence disease progression but also affect immunotherapy efficacy (63). Therefore, an in-depth understanding of the functional characteristics and regulatory mechanisms of ActMono might provide important clues for the development of new therapeutic strategies.

This study, through the integration of multiple single-cell datasets, provides a comprehensive understanding of tumour cell functional characteristics during CRC progression and their effects on the immune microenvironment, particularly emphasizing the important role of the SecTA subgroup in tumour progression through the regulation of multiple mechanisms, including proliferation, apoptosis, and microenvironment remodelling. We found that ActMono, an important endpoint state of myeloid cells, not only is significantly enriched in tumour tissue but also plays a key role in connecting innate and adaptive immune responses through regulating T-cell function, suggesting its important role in shaping the tumour immune microenvironment. These findings provide important clues for understanding the immune escape mechanisms of CRC and developing new therapeutic strategies. Besides, through machine learning algorithms, we have established a CRS system that demonstrates outstanding prognostic performance. Meanwhile, there have been no previous studies on the role of *TUBB* in colorectal cancer. Our study, for the first time, reveals the role of *TUBB* in CRC progression by combining bioinformatics with clinical sample analysis, emphasizing its potential as a significant risk factor and we also validated its proliferation, anti-apoptosis, and metastasis ability.

Despite the comprehensive nature of our integrative single-cell analysis and the identification of key cellular subpopulations such as SecTA and ActMono, we acknowledge several limitations in this study. First, our findings rely heavily on in silico analyses, and although we have validated TUBB expression and function using patient samples and *in vitro* assays, the downstream signalling pathways regulated by TUBB and the mechanistic interdependencies between SecTA, ActMono, and immune modulation remain to be experimentally elucidated. Future studies utilizing *in vivo* models and perturbation assays, such as gene editing or pathway inhibition, will be necessary to unravel the causative roles of these interactions. Second, technical limitations inherent to single-cell RNA sequencing, such as dropout events and batch effects, may affect gene expression quantification and cell type annotation. Although we applied established correction and integration methods, residual biases may persist. Third, while the CRC Risk Score (CRS) prognostic model demonstrated good performance in internal validation, its generalizability remains to be confirmed. External validation using independent CRC cohorts and cross-validation across additional publicly available datasets are needed to further assess the model’s robustness and minimize the risk of overfitting. Lastly, although we integrated 41 samples from four datasets, the sample size is still relatively limited. Future studies with larger, more diverse patient cohorts and multi-omics integration will be critical to validate and extend our findings, and to explore the translational potential of SecTA, ActMono, and TUBB as therapeutic targets or biomarkers.

## Data Availability

The original contributions presented in the study are included in the article/**Supplementary Material**. Further inquiries can be directed to the corresponding authors.
