# Supplementary material for: Single-cell sequencing reveals dysregulated cell type perturbations and critical mediator communication remodelling in colorectal cancer
Source: Front Immunol. 2025 Jun 5;16:1557564. doi: 10.3389/fimmu.2025.1557564 (PMC12176898; doi:10.3389/fimmu.2025.1557564)
Supplement: Supplementary file 1 [file Table1.docx]

| **Supplementary Table 1. Cell Annotation Marker Genes** | |
| --- | --- |
| Marker genes for each subpopulation used for cell annotation | |
|  |  |
| **Cell type** | **Markers** |
| T lymphocytes (Tcells) | CD3D, CD3E, TRAC |
| B lymphocytes (Bcells) | CD79A, MS4A1,CD79B |
| Epithelial cells (Epi) | EPCAM, KRT8, KRT18 |
| Plasma cells (Plasma) | JCHAIN, SDC1 |
| Myeloid cells (Mye) | LYZ, MNDA, C1QA |
| Fibroblasts (Fibro) | COL1A1, DCN, COL1A2 |
| Endothelial cells (Endo) | CLDN5, CDH5, PECAM1, VWF |
| Mast cells (MAST) | TPSB2, TPSAB1 |


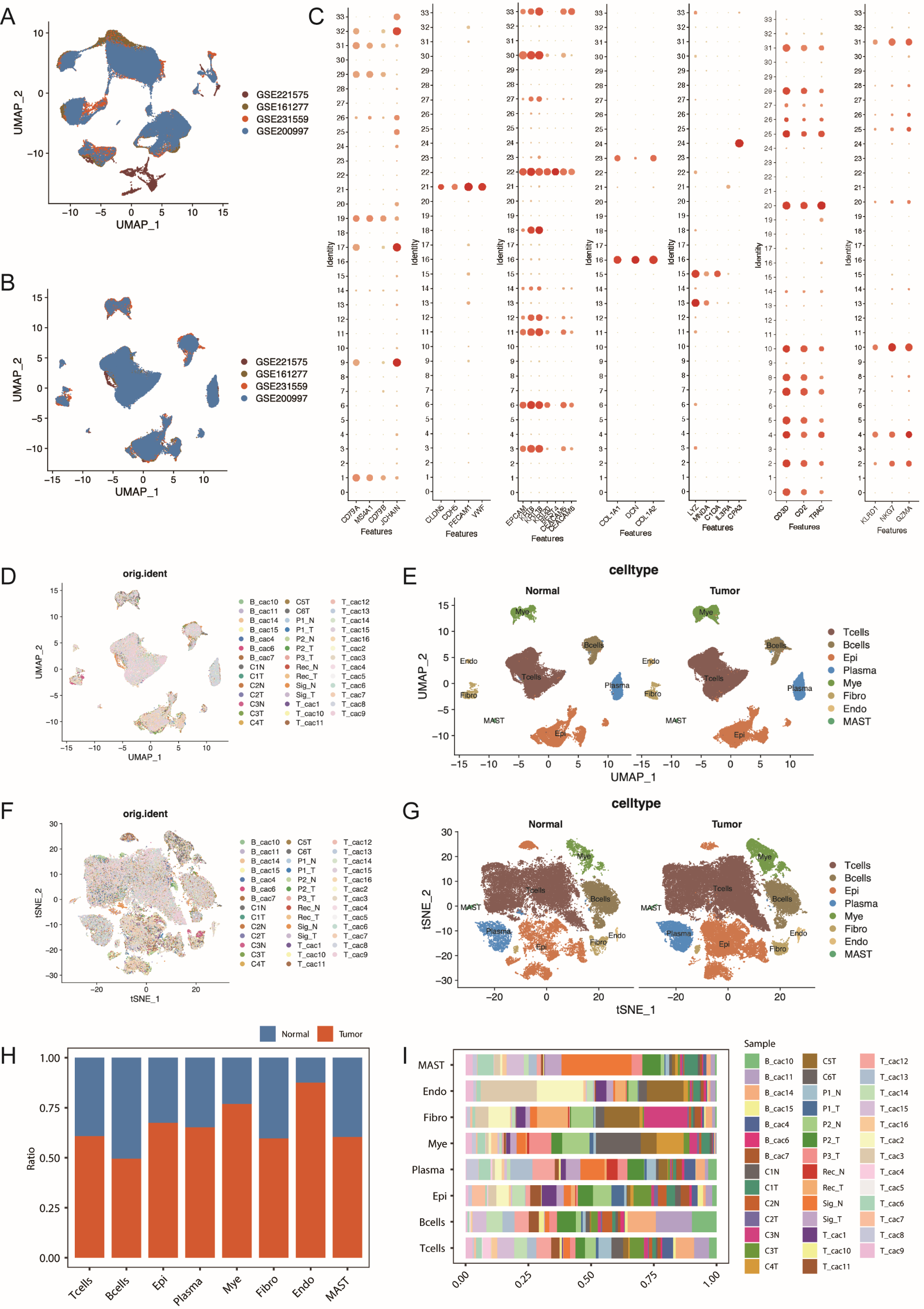


Figure S1: (A-B) UMAP comparison before and after batch effect correction. (C) Annotation of different clusters using typical marker genes. (D-E) UMAP plots showing the sample origin of each cell, with UMAP distribution of each cell type displayed by group. (F-G) t-SNE plots showing the sample origin of each cell, with t-SNE distribution of each cell type displayed by group. (H-I) Stacked bar plots displaying the proportions of (H) groups and (I) sample origins for each cell type.


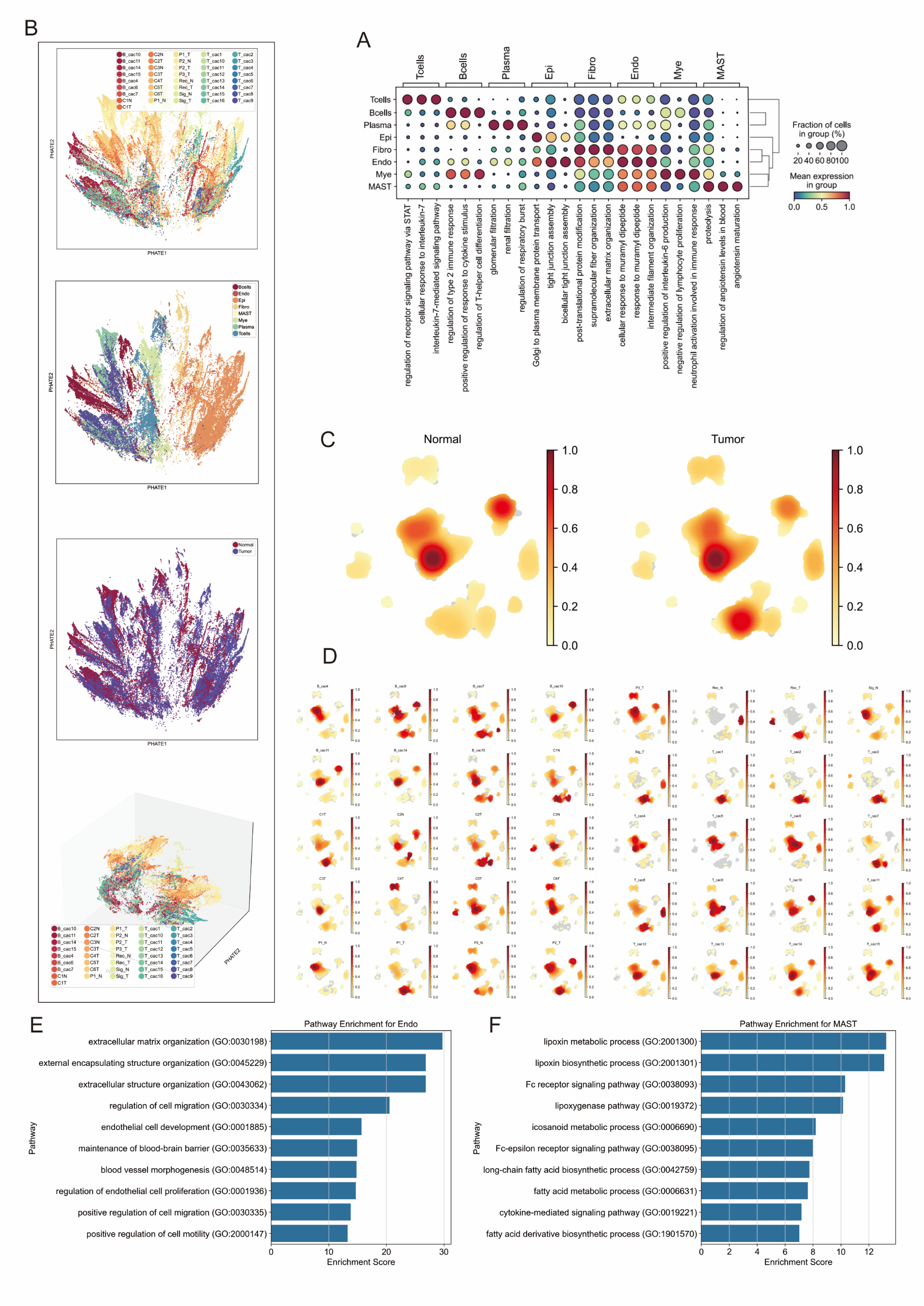


Figure S2: (A) AUCell analysis showing the top 3 specifically activated pathways for each cell type. (B) Two-dimensional visualization of PHATE results, colored by sample origin, cell type, and group, alongside a three-dimensional visualization of PHATE results colored by sample origin. (C) Density plot showing the enrichment of cell counts in each group. (D) Density plot showing cell abundance for each sample. (E-F) Top 10 specifically activated pathways for Endo and MAST cell types.


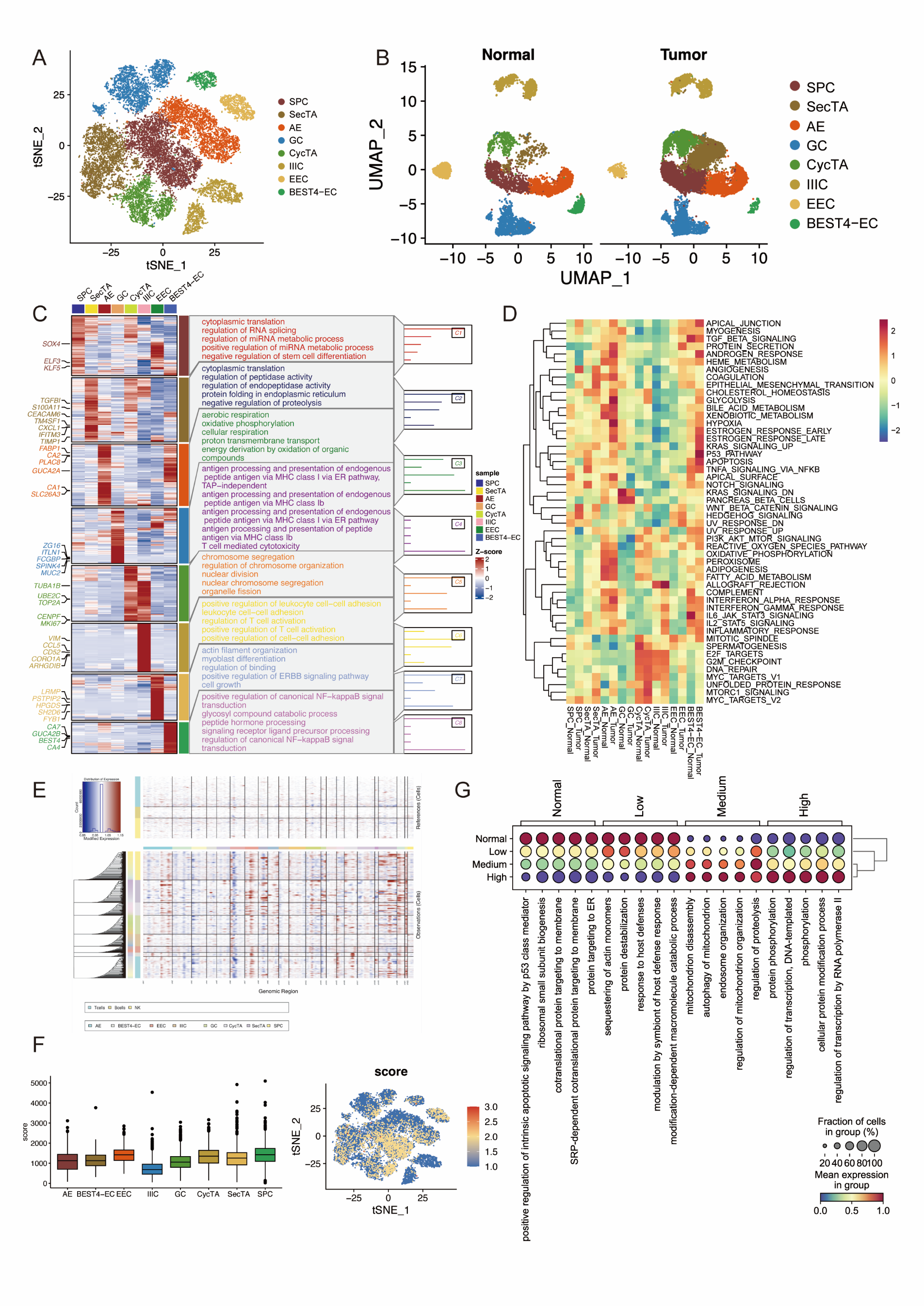


Figure S3: (A) t-SNE plot showing the distribution of epithelial cell subtypes. (B) UMAP plots displaying epithelial cell subtypes by group. (C) Left: Dynamic changes in representative differentially expressed genes (DEGs) across malignant cell populations. Middle: Heatmap showing representative DEGs between each cell group. Right: Representative enriched Gene Ontology (GO) terms for each cluster. (D) GSVA scores of epithelial cell subtypes, calculated separately for tumor and normal groups. (E) InferCNV results before noise filtering. (F) CNV scores of epithelial cell subtypes and t-SNE visualization of these scores. (G) Top 5 activated pathways in epithelial cells with different CNV statuses.


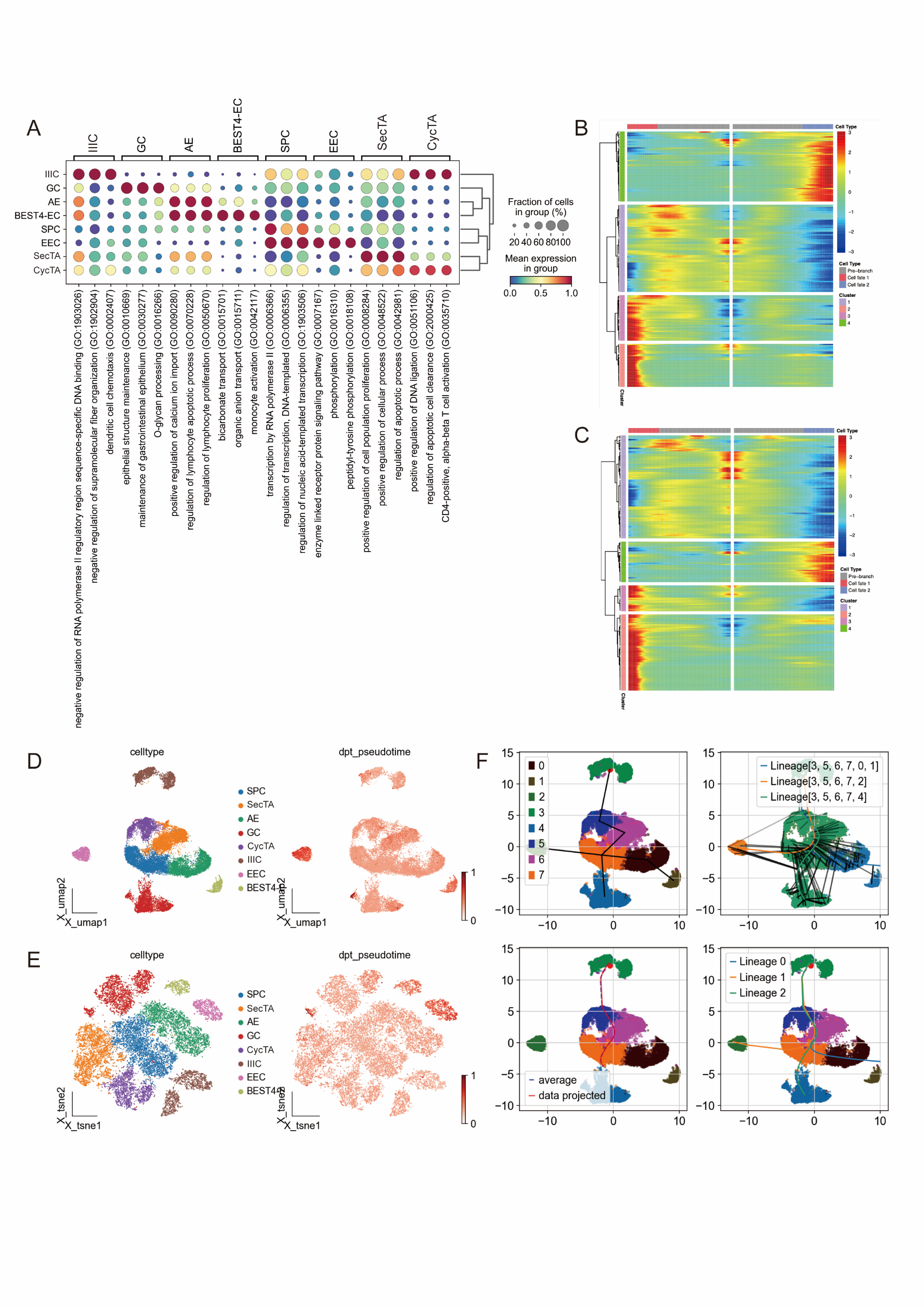


Figure S4: (A) AUCell analysis showing the top 3 specifically activated pathways for each epithelial cell subtype. (B-C) Pseudotime heatmaps showing the top 200 genes identified by BEAM analysis before and after nodes 1 and 2. (D-E) PAGA pseudotime results visualized by UMAP and t-SNE. (F) Slingshot analysis identifying potential lineages among epithelial cell subtypes.


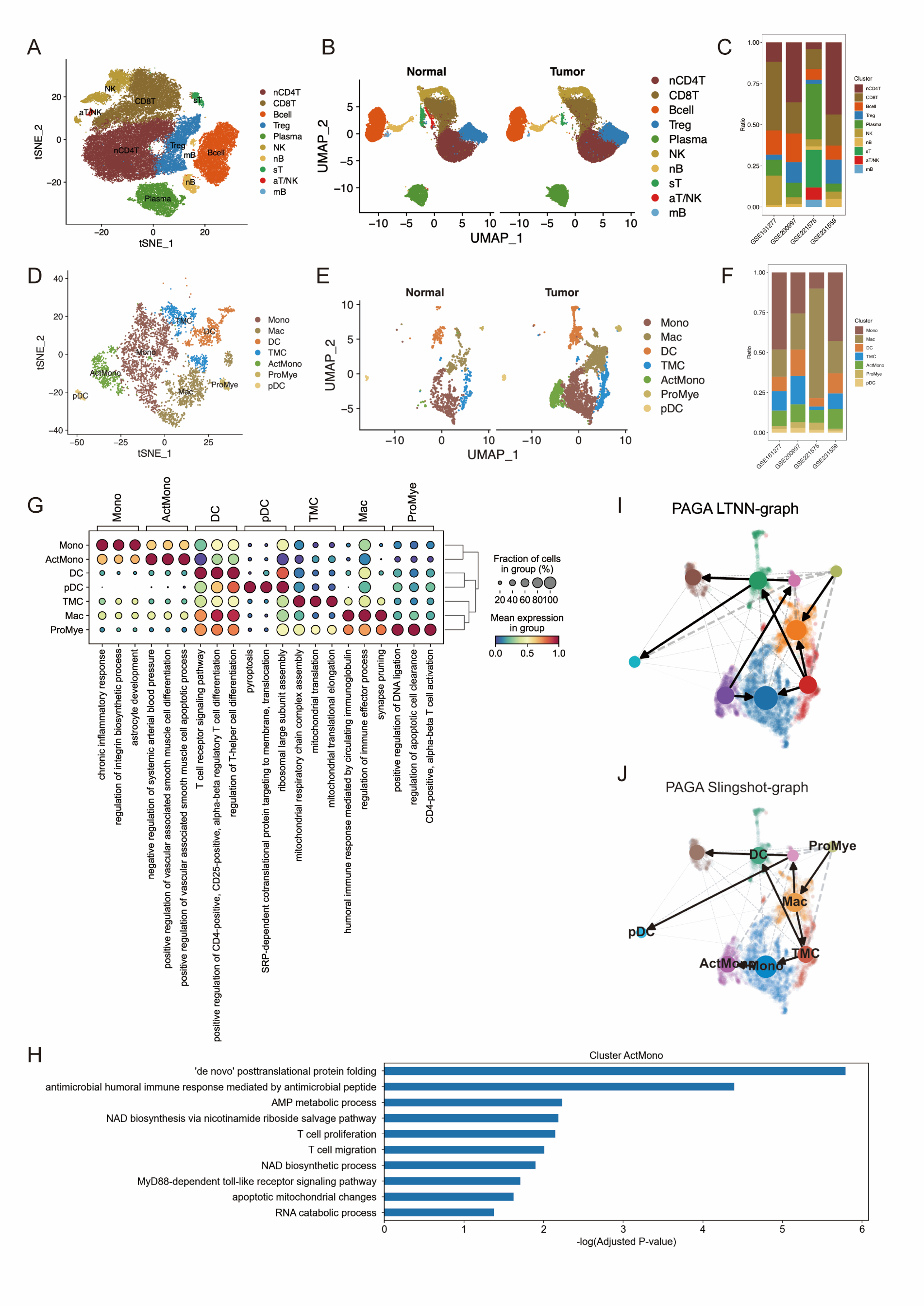


Figure S5: (A) t-SNE plot showing the distribution of all cells. (B) UMAP plots displaying lymphoid cell types by group. (C) Proportions of cell types in each dataset. (D) t-SNE plot showing the distribution of all cells. (E) UMAP plots displaying myeloid cell types by group. (F) Proportions of cell types in each dataset. (I) AUCell analysis showing the top 3 specifically activated pathways for each myeloid cell subtype. (J) Top 10 pathways specifically activated in ActMono. (G-H) Trajectory inference using PAGA, and integrated trajectory inference results combining PAGA and Slingshot.


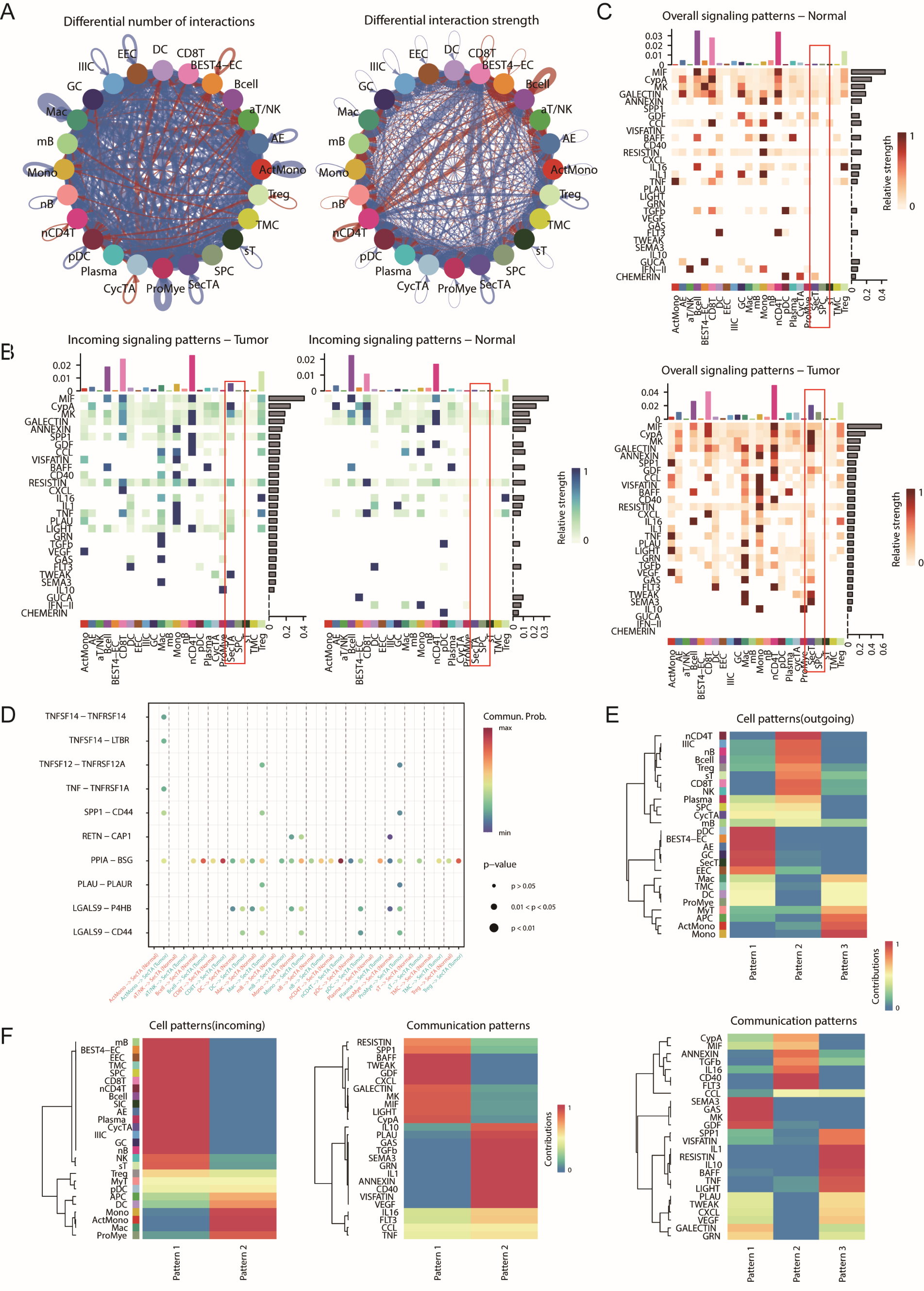


Figure S6: (A) Cell communication network analysis of epithelial and immune cell subtypes, with networks displaying the number and strength of communications. (B) Heatmap showing incoming signal strength for each cell type in the Tumor and Ctrl groups. (C) Heatmap showing total signal strength for each cell type in the Tumor and Ctrl groups. (D) Bubble plot showing receptor-ligand pairs with significant differences between the Tumor and Normal groups, with SecTA as the signal receiver. (E-F) Pattern recognition in outgoing and incoming signaling modes.


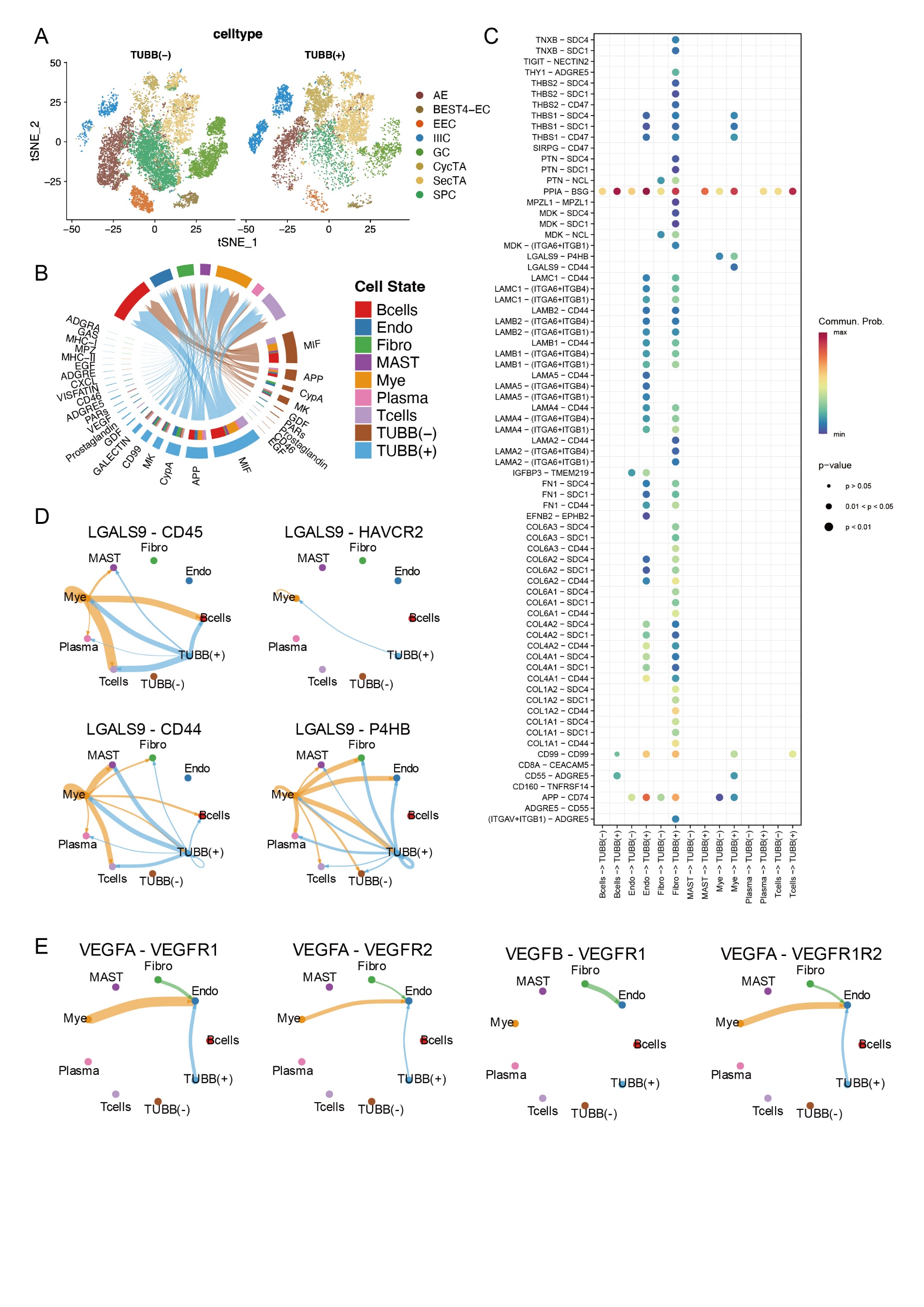


Figure S7: (A) t-SNE visualization of TUBB (+) and TUBB (-) epithelial cells. (B) Primary signals received by TUBB (+) and TUBB (-) cells as signal senders. (C) Bubble plot showing interactions between TUBB (+) and TUBB (-) epithelial cells as signal receivers and immune cells. (D-E) The communication diagram shows the specific receptor-ligand pairs of TUBB(+) and TUBB(-) in LGALS9 and VEGF-related signaling pathways.


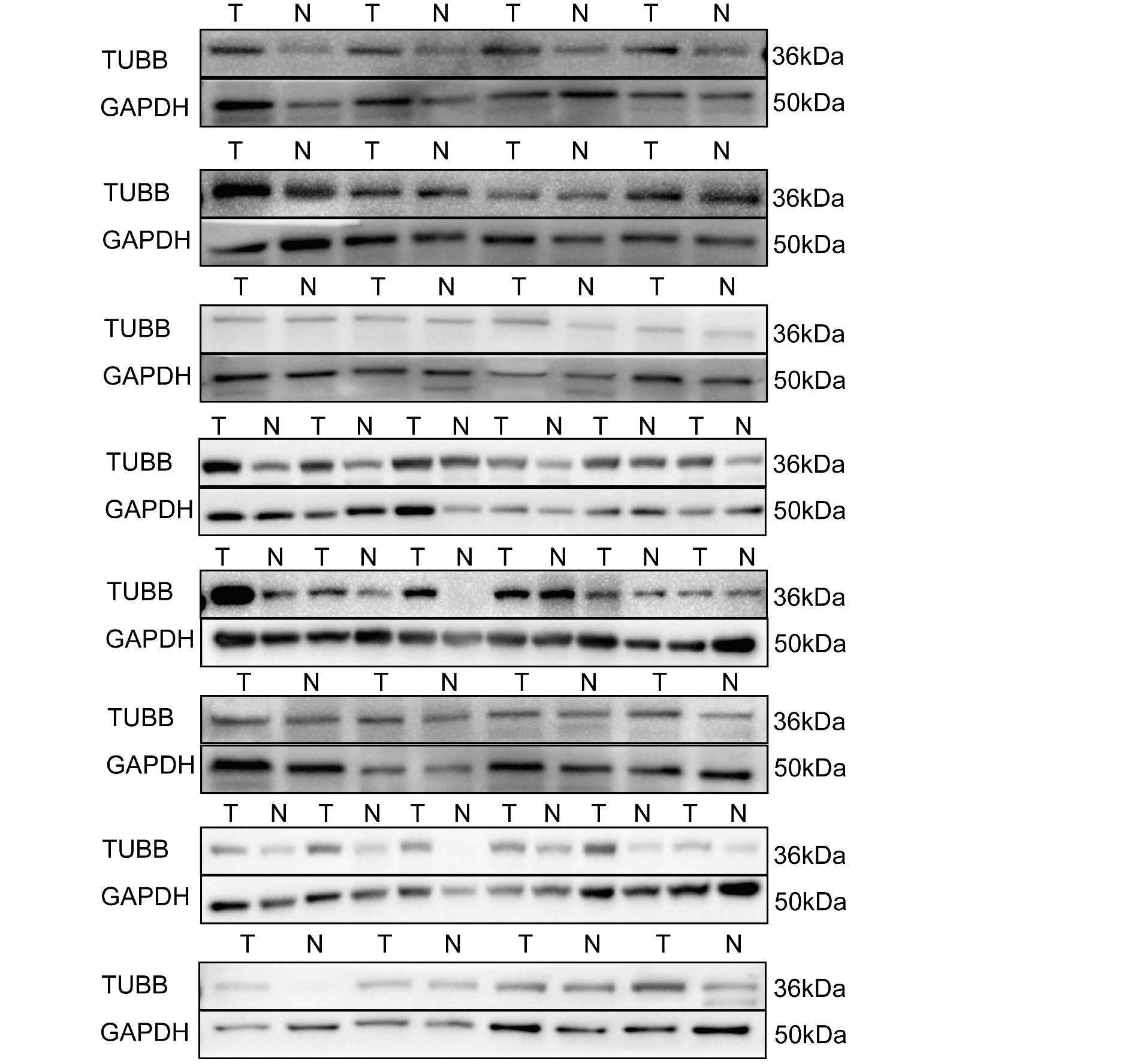


Figure S8: The protein expression of TUBB in 38 pairs of tissues.

Full-length blots/gels are presented in Additional file 2
