# Supplementary figures and images for: Single-cell sequencing reveals dysregulated cell type perturbations and critical mediator communication remodelling in colorectal cancer
Source: Front Immunol. 2025 Jun 5;16:1557564. doi: 10.3389/fimmu.2025.1557564 (PMC12176898; doi:10.3389/fimmu.2025.1557564)

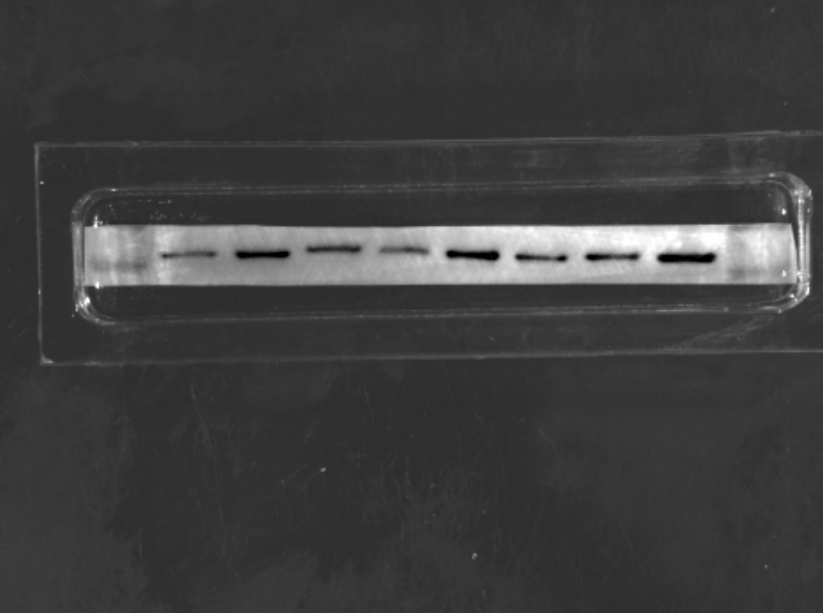

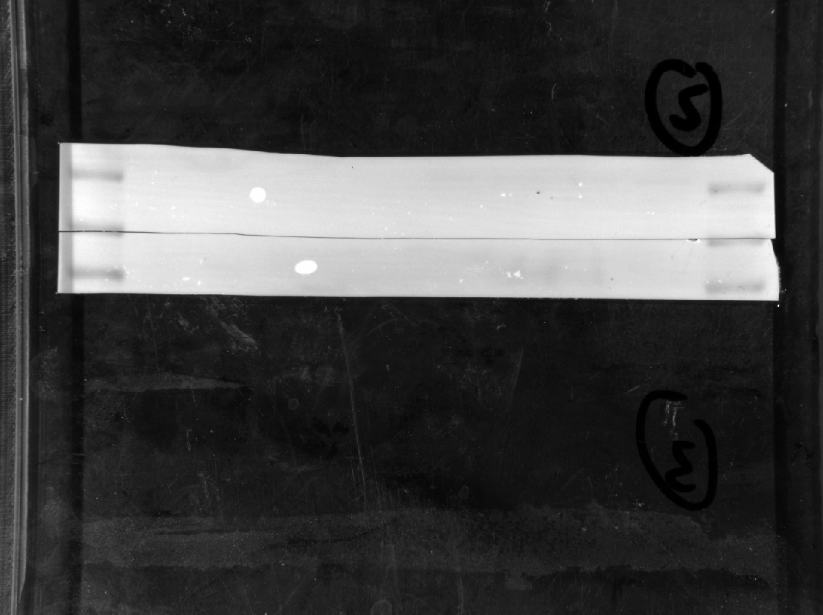

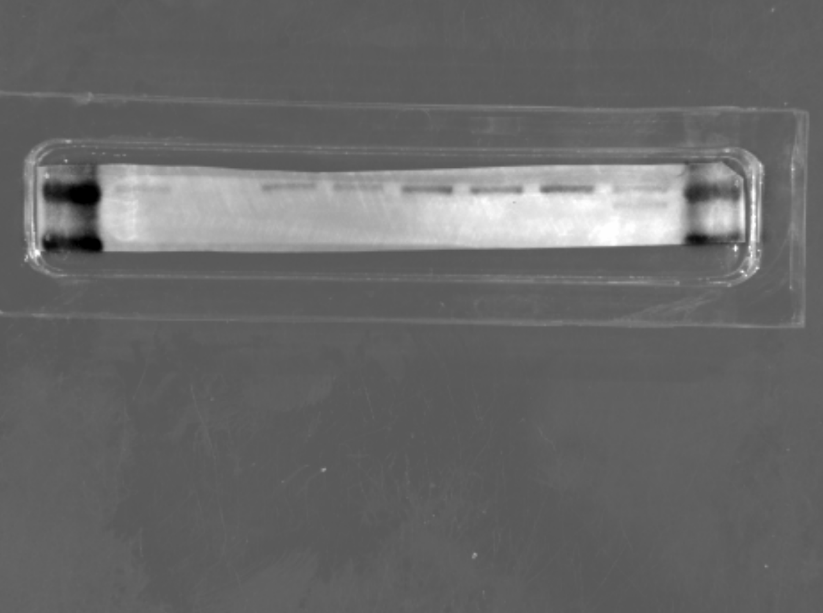


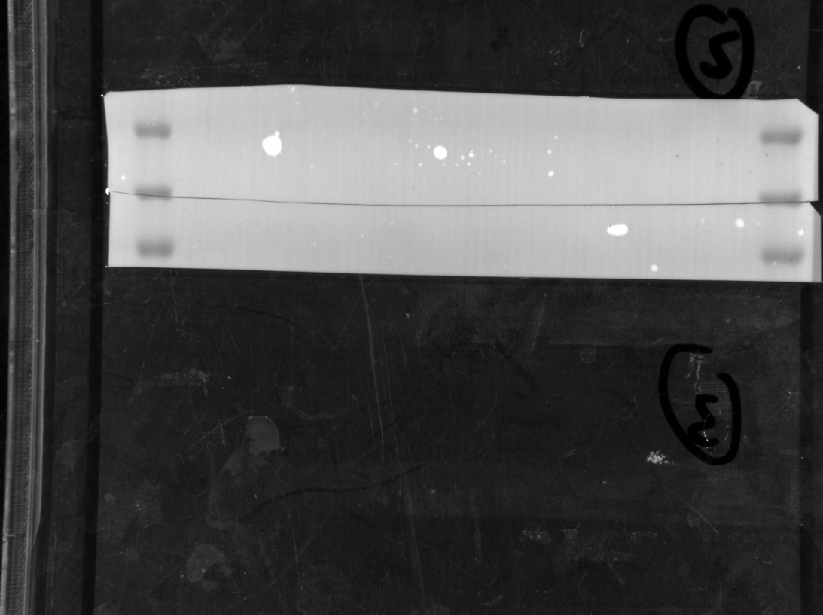

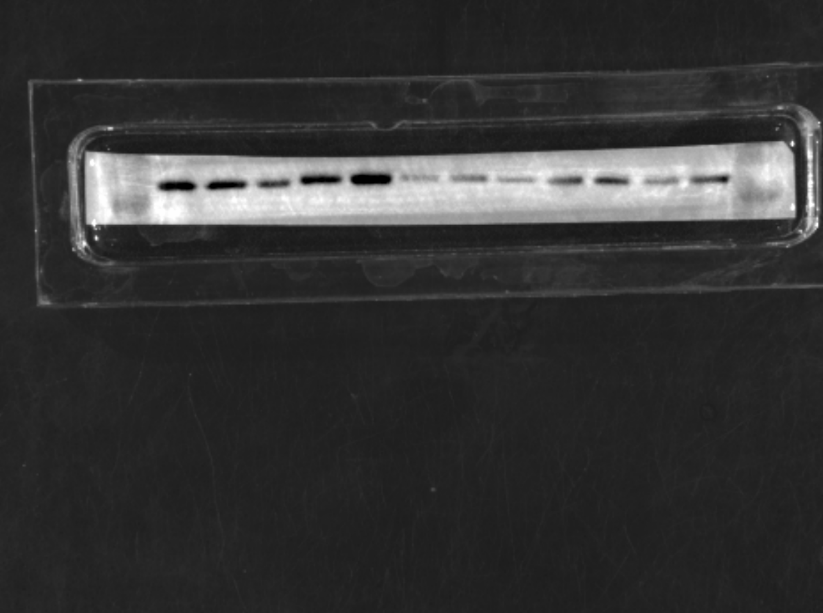

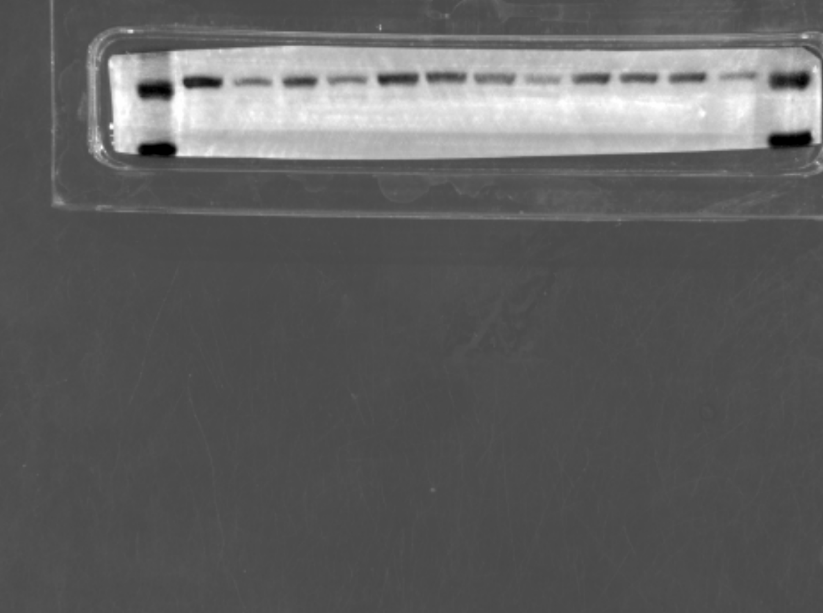


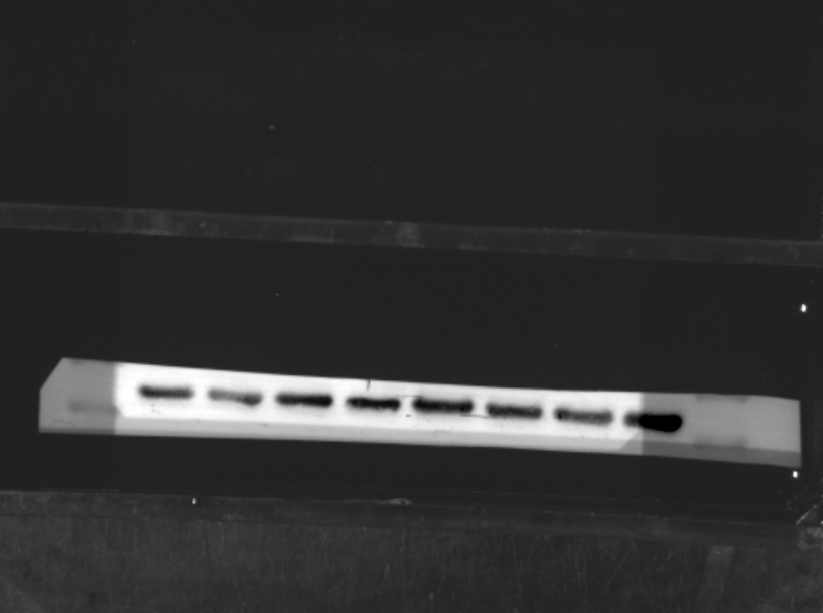

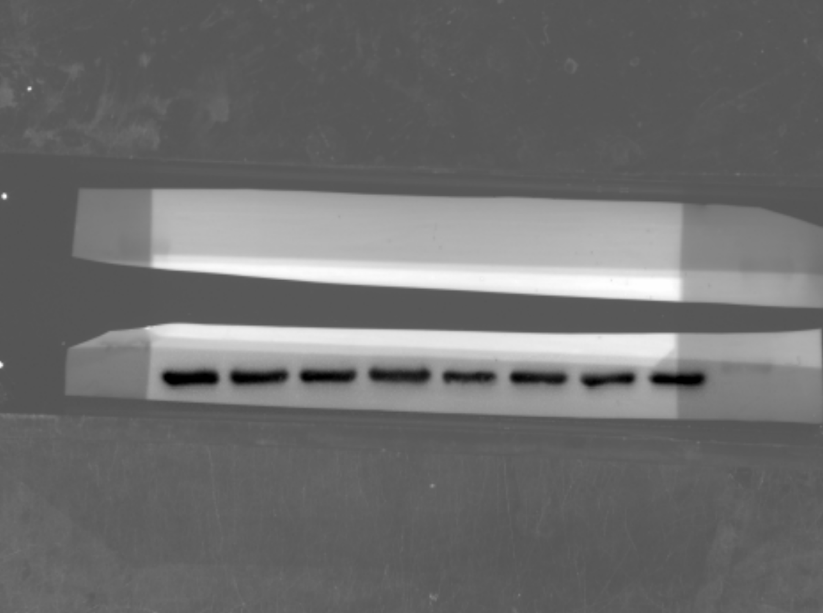

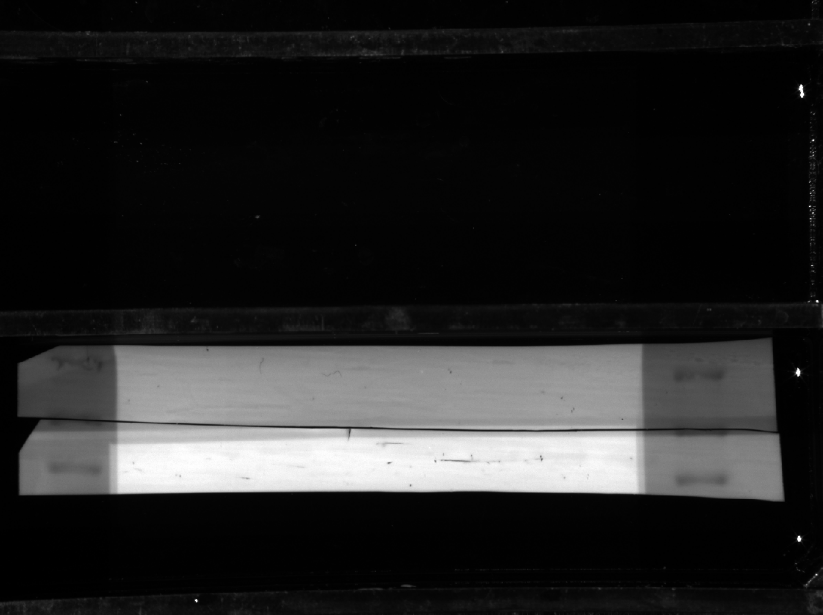


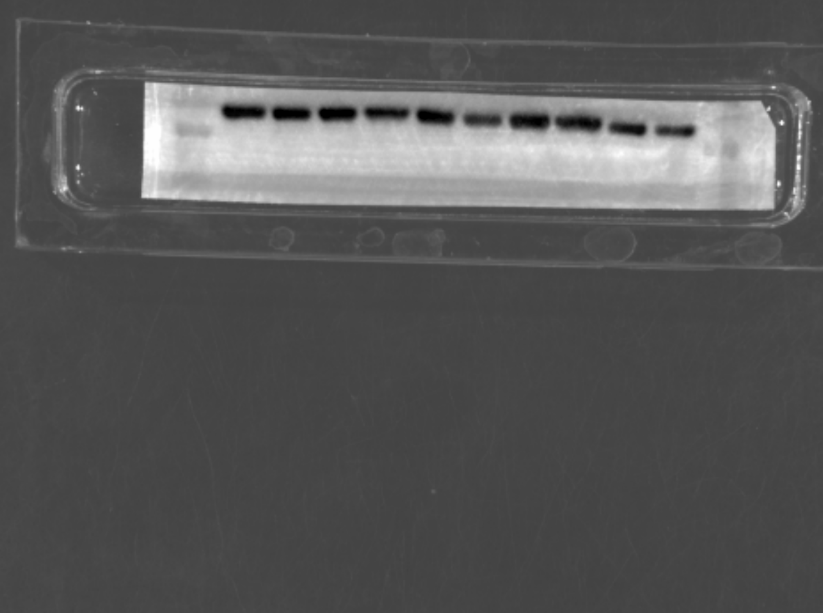


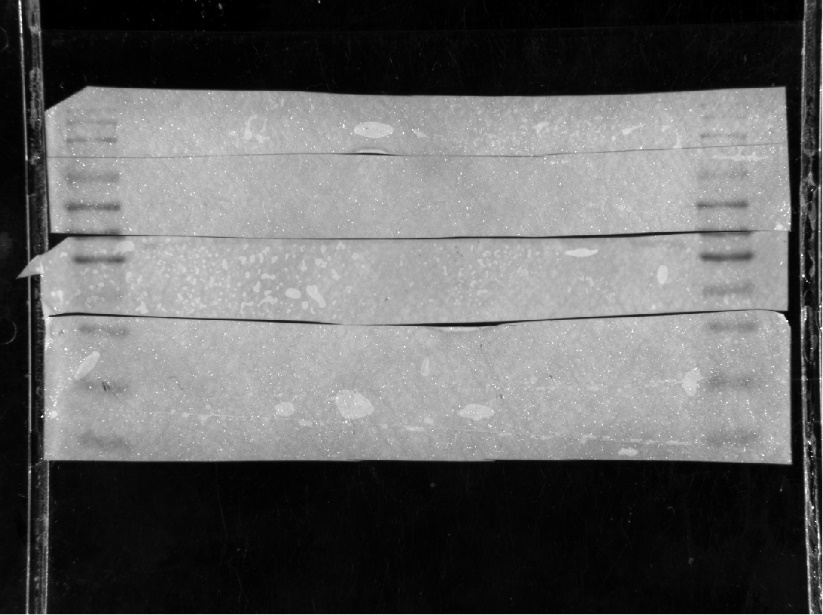

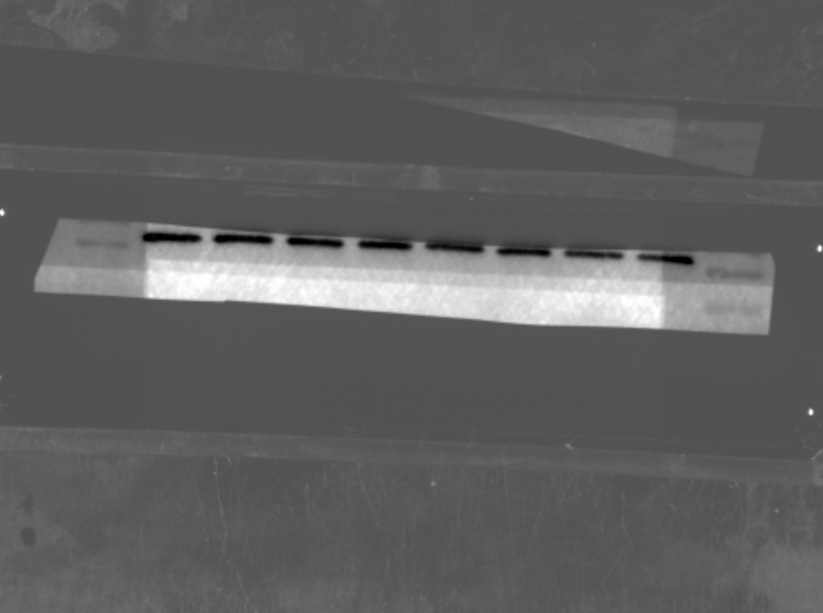

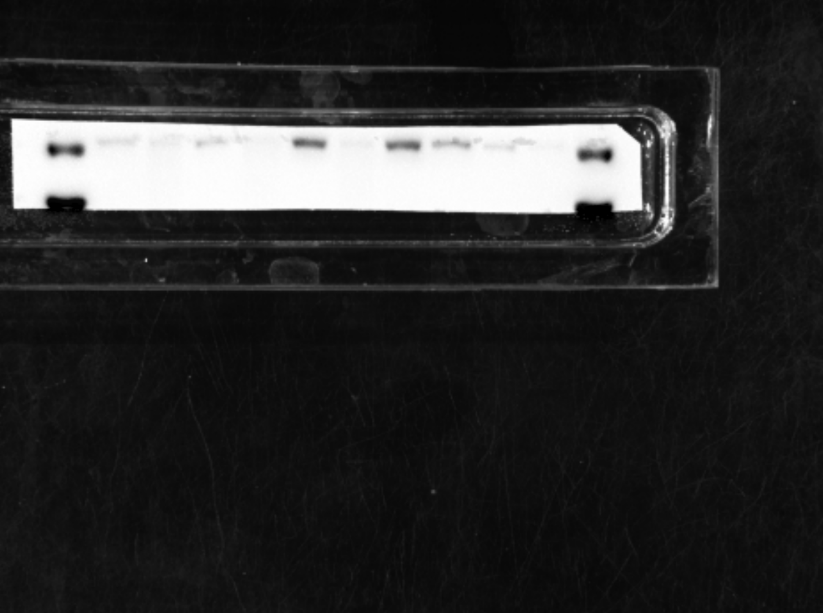

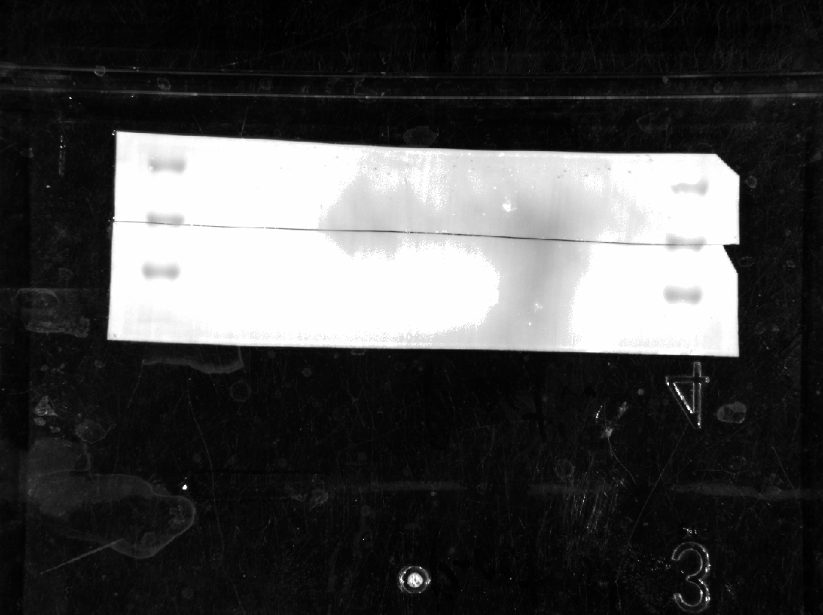


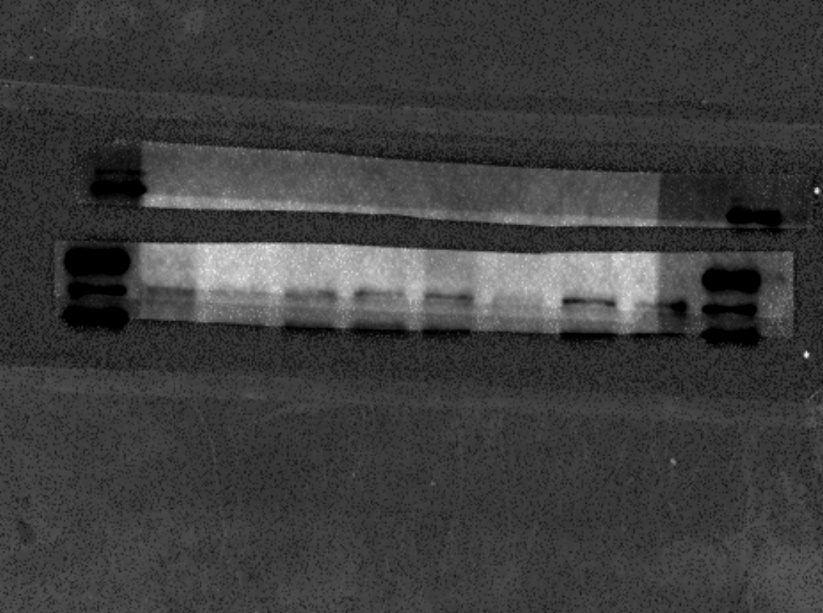


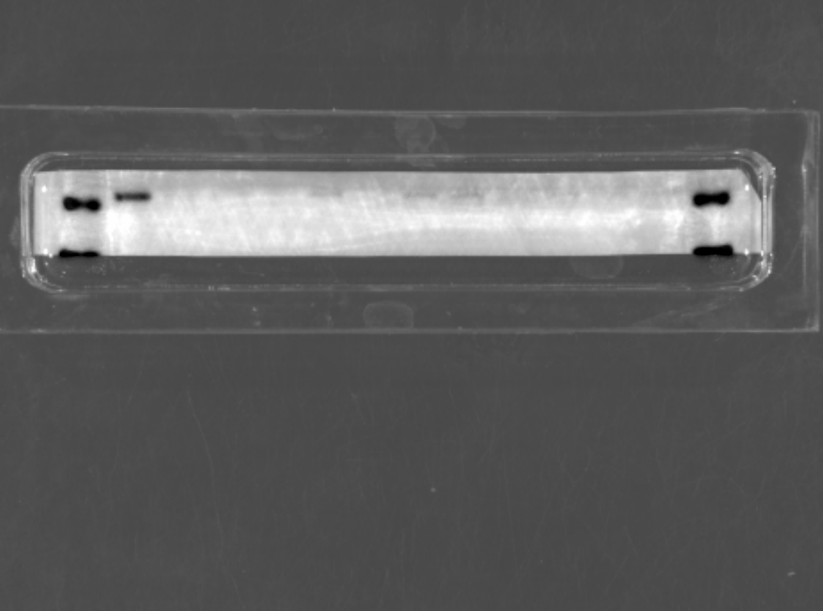

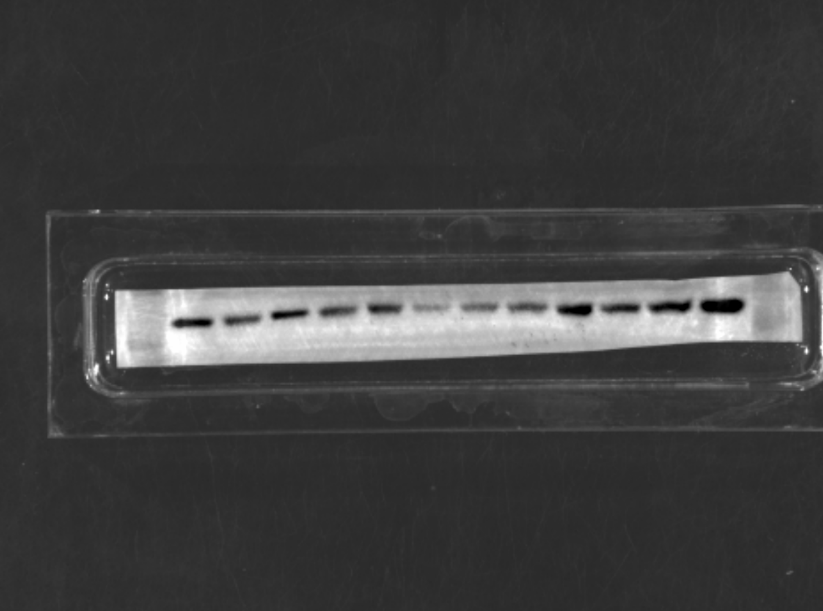

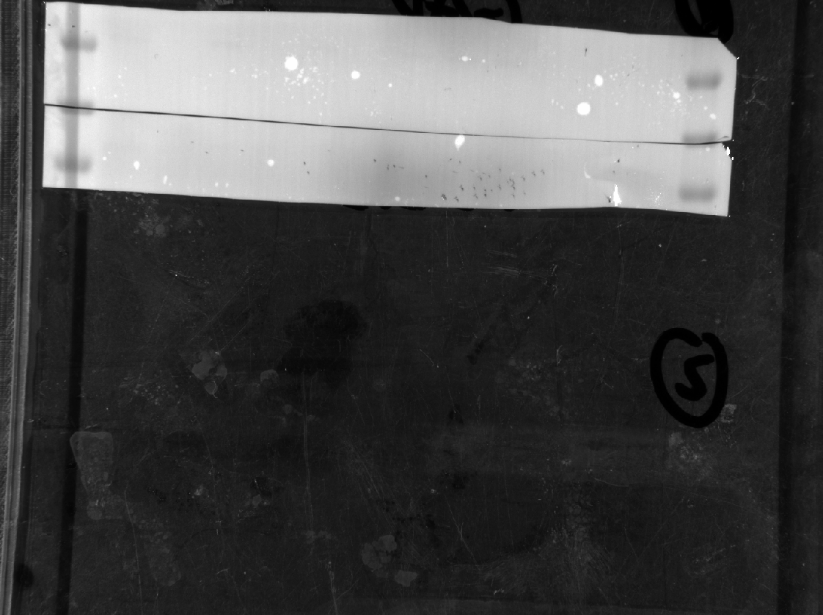

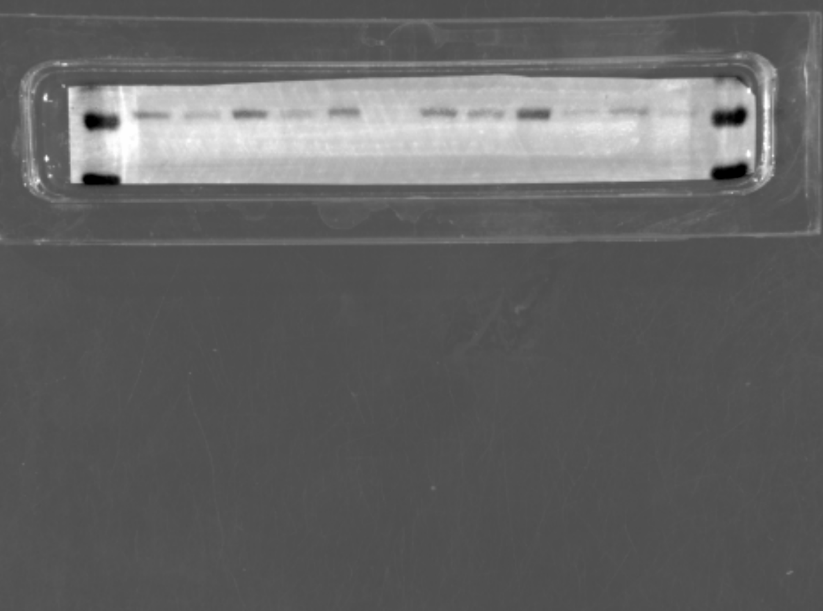

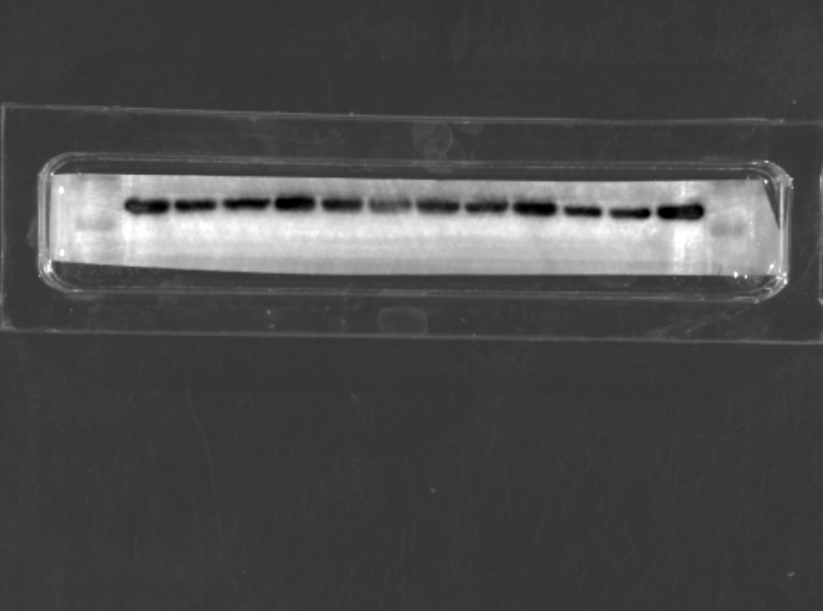


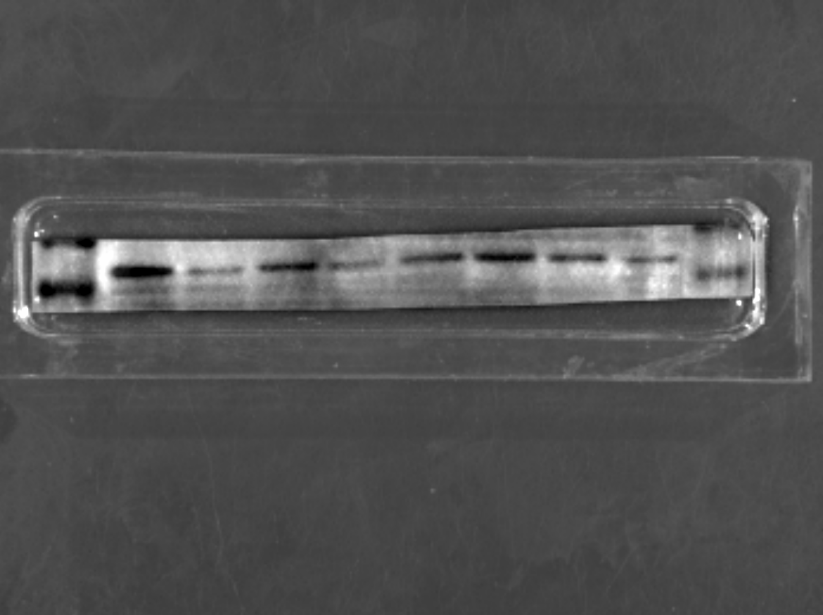

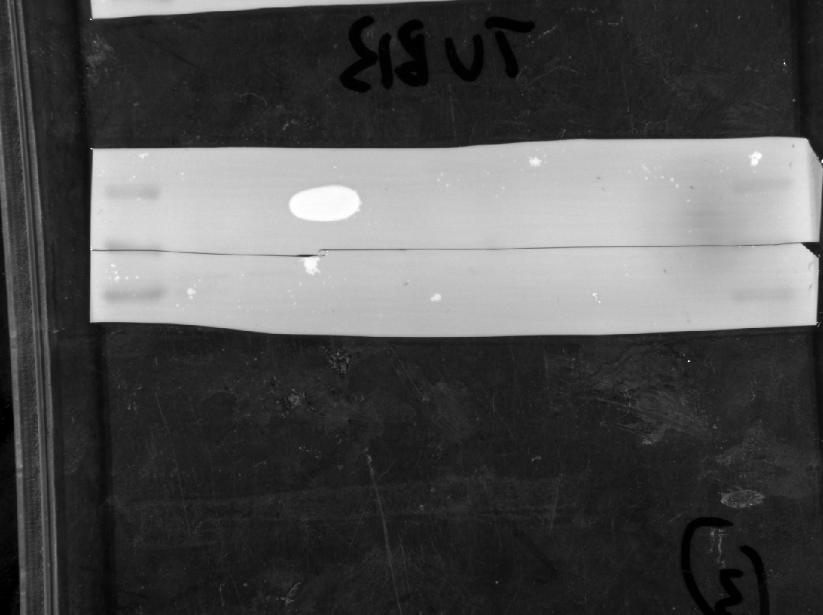

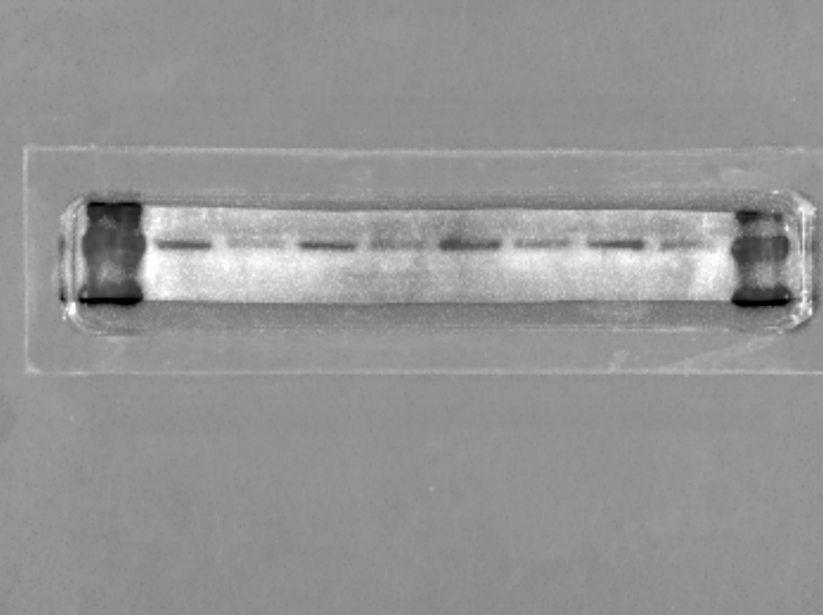

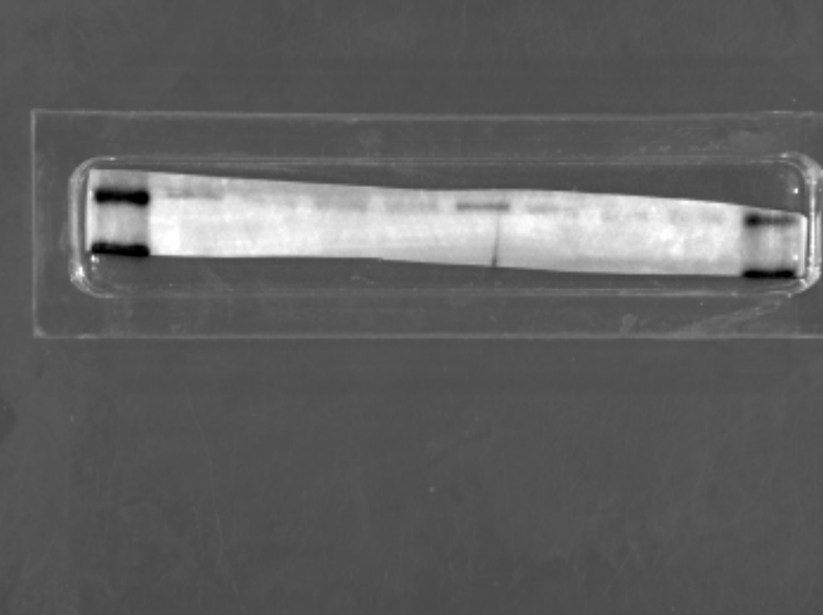

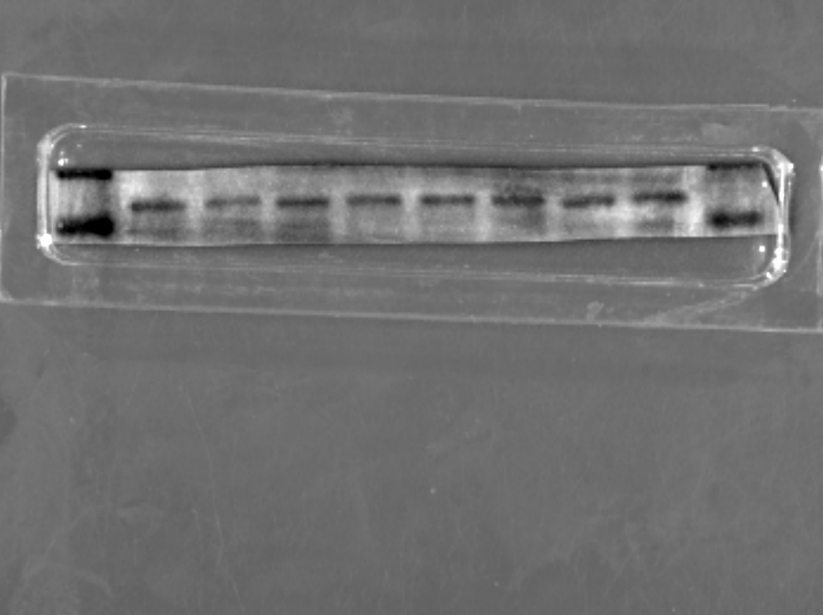

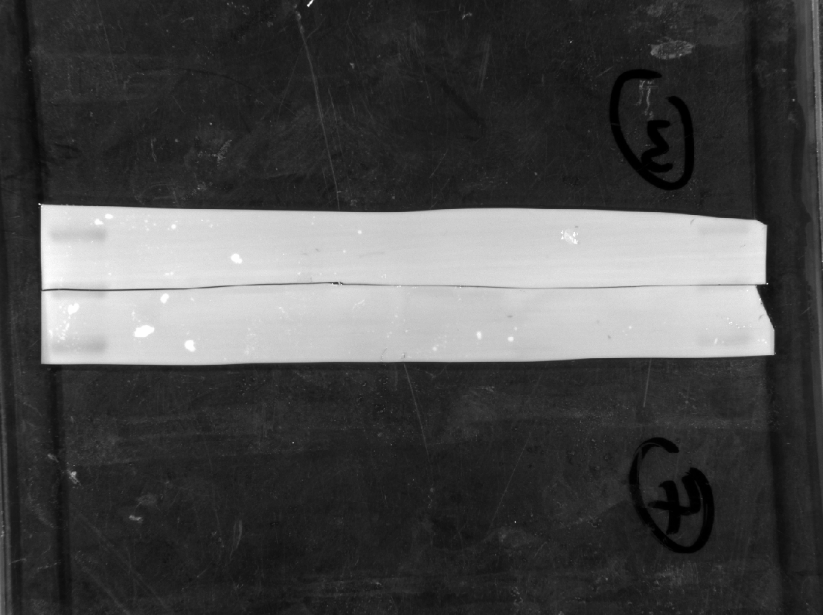


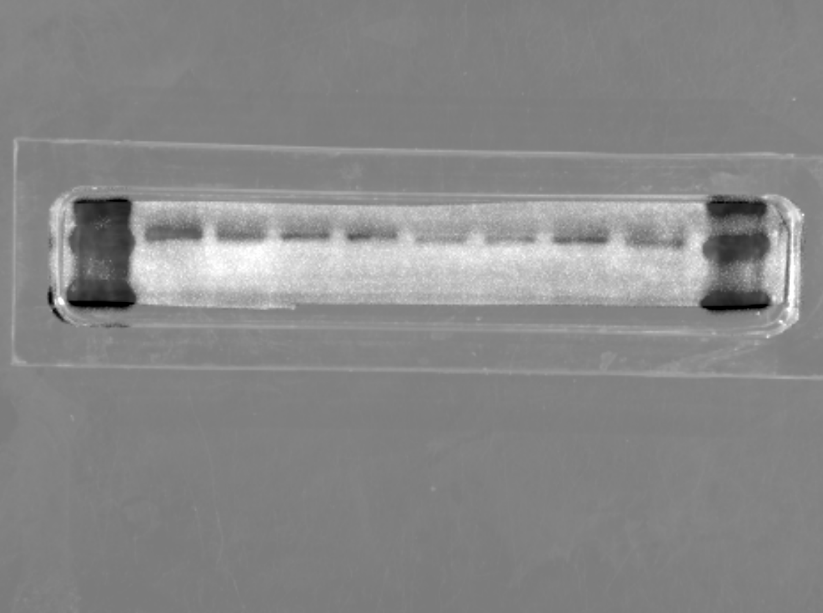

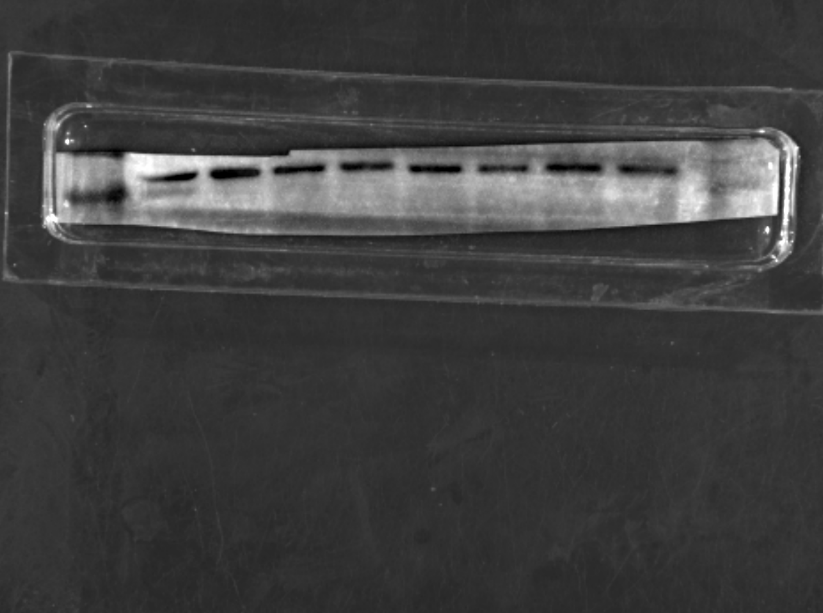


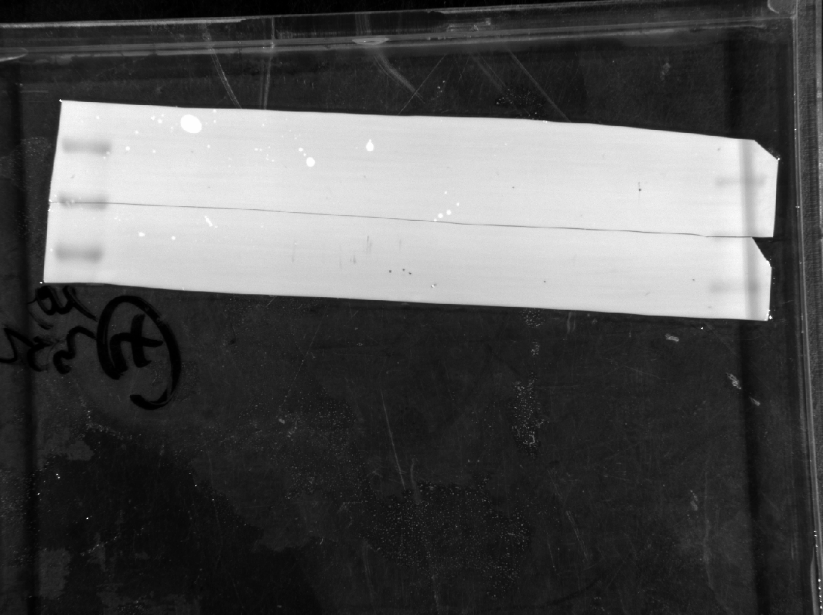

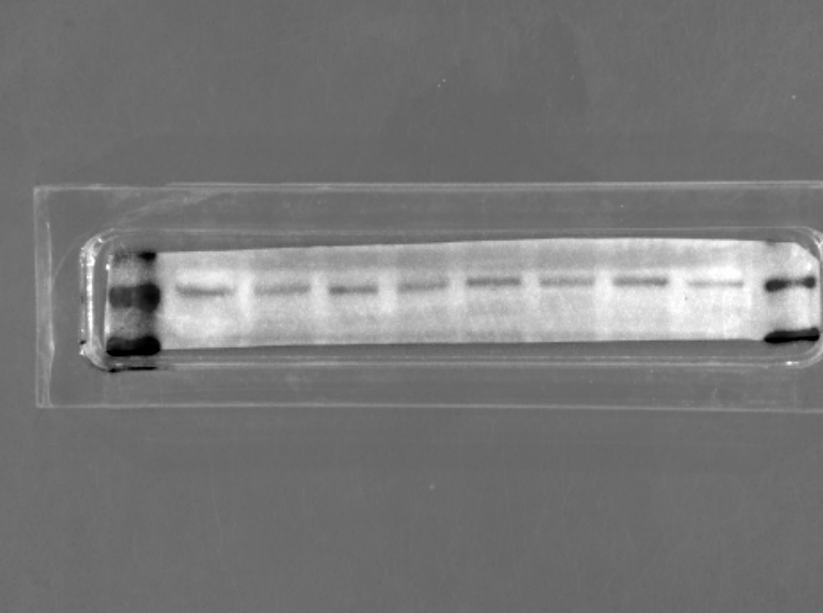

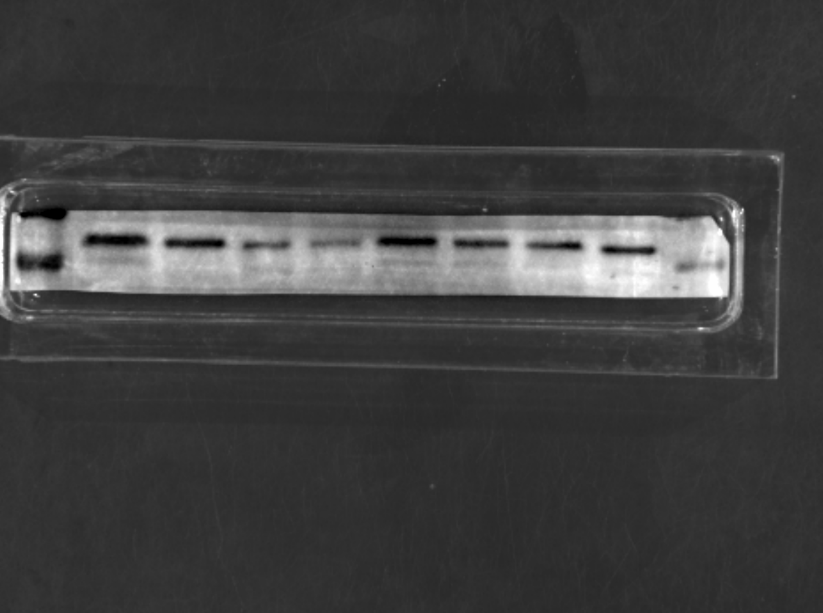


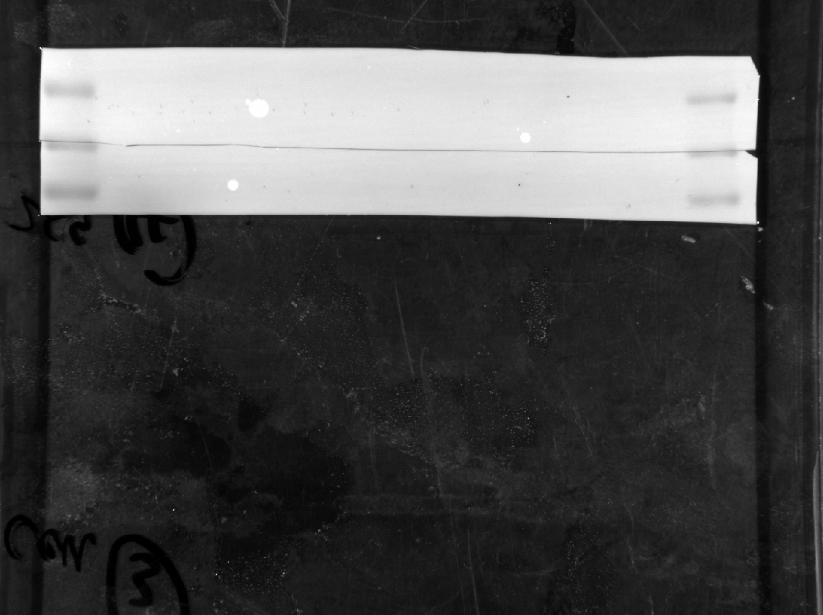

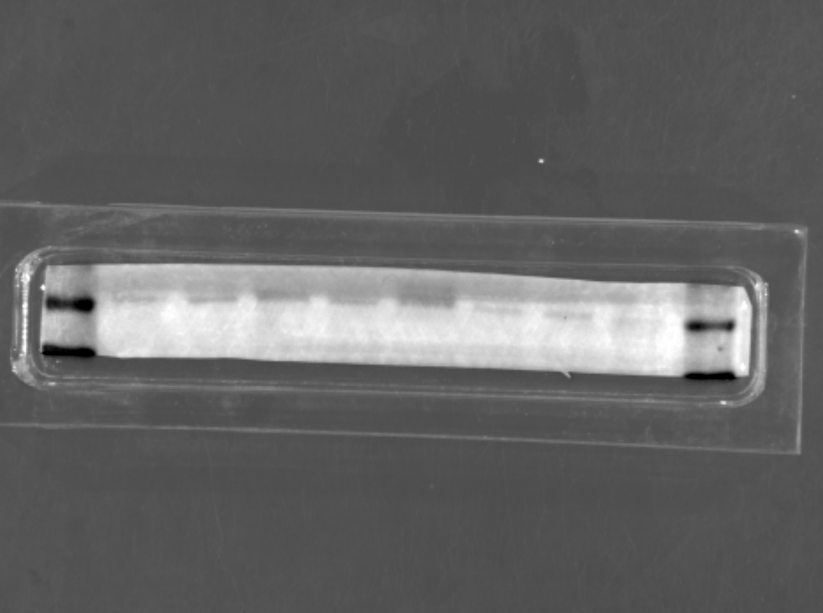

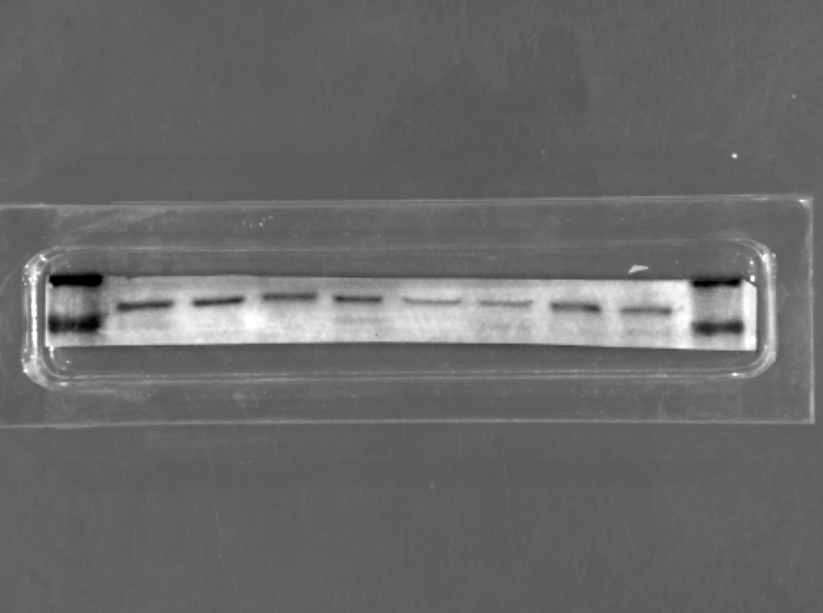


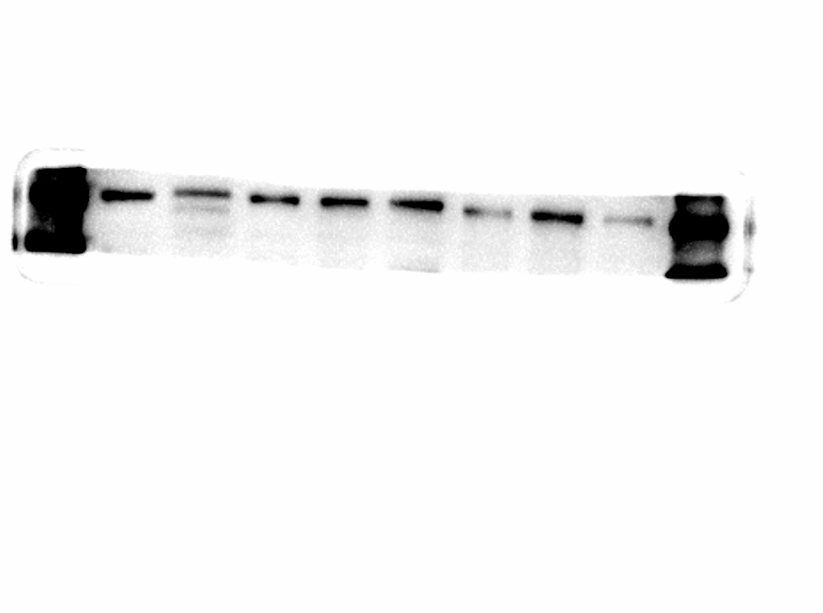

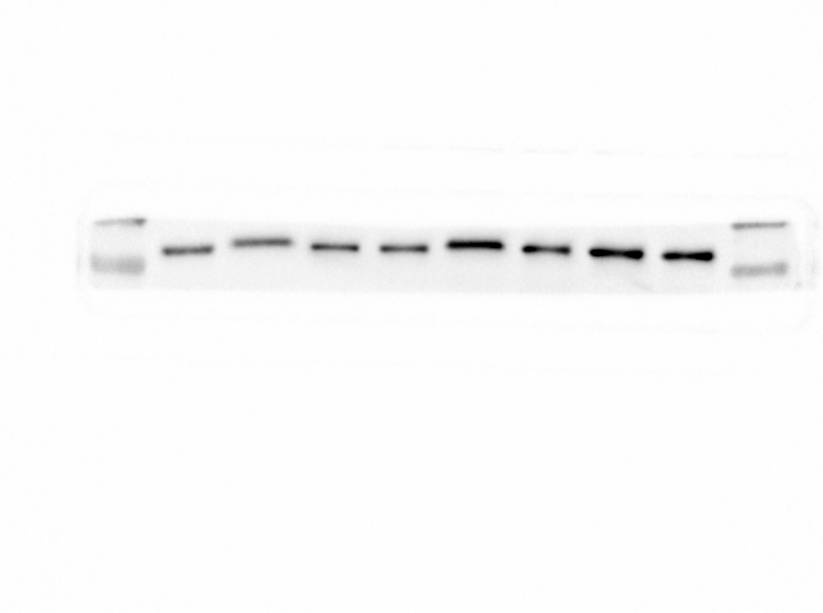

Supplement: Supplementary file 2 [file Table2.docx]
